# One step engineering of the small-subunit ribosomal RNA using CRISPR/Cas9

**DOI:** 10.1038/srep30714

**Published:** 2016-08-04

**Authors:** Krishna Kannan, Billyana Tsvetanova, Ray-Yuan Chuang, Vladimir N. Noskov, Nacyra Assad-Garcia, Li Ma, Clyde A. Hutchison III, Hamilton O. Smith, John I. Glass, Chuck Merryman, J. Craig Venter, Daniel G. Gibson

**Affiliations:** 1 Synthetic Genomics, Inc., La Jolla, CA 92037, USA; 2 J. Craig Venter Institute, La Jolla, CA 92037, USA.

## Abstract

Bacteria are indispensable for the study of fundamental molecular biology processes due to their relatively simple gene and genome architecture. The ability to engineer bacterial chromosomes is quintessential for understanding gene functions. Here we demonstrate the engineering of the small-ribosomal subunit (16S) RNA of *Mycoplasma mycoides*, by combining the CRISPR/Cas9 system and the yeast recombination machinery. We cloned the entire genome of *M. mycoides* in yeast and used constitutively expressed Cas9 together with *in vitro* transcribed guide-RNAs to introduce engineered 16S rRNA genes. By testing the function of the engineered 16S rRNA genes through genome transplantation, we observed surprising resilience of this gene to addition of genetic elements or helix substitutions with phylogenetically-distant bacteria. While this system could be further used to study the function of the 16S rRNA, one could envision the “simple” *M. mycoides* genome being used in this setting to study other genetic structures and functions to answer fundamental questions of life.

The Clustered Regularly Interspaced Short Palindromic Repeats (CRISPR) and the CRISPR Associated Systems (Cas) are native to bacteria and archaea, which provide adaptive immunity against invading nucleic acids such as those from viruses^1^,^2^. However, the RNA-mediated nuclease activity of one of these systems found in *Streptococcus pyogenes* that creates double-stranded breaks against foreign DNA has been exploited for precise and scar-free genome editing applications in both bacteria and higher eukaryotes^3^,^4^,^5^,^6^,^7^,^8^,^9^,^10^,^11^,^12^,^13^,^14^. While fewer studies have delineated the use of this system in bacteria, it has been extensively applied in yeast and higher eukaryotes^8^,^9^,^10^,^11^,^12^,^13^,^14^. Due to the lack of efficient homologous recombination machineries in bacteria such as *Escherichia coli*, genome editing with CRISPR/Cas9 usually needs to be accompanied by proteins from viruses such as the ones that constitute the lambda-Red or the Rac prophage recombination systems^8^,^9^,^10^,^11^,^12^,^13^,^14^, thus limiting the applicability of this type of genome-engineering to few bacteria. However, homologous recombination is efficiently accomplished in conjunction with the CRISPR/Cas9-targeted cleavage by native machineries present in the eukaryotic chromosomes^3^,^4^,^5^,^6^,^7^, without the need for heterologous proteins.

Bacteria such as *E. coli* have traditionally been used as model organisms to study and understand the fundamental processes in biology such as replication, transcription and translation, due to the simplicity in their genome architecture and tractable genetics. Targeted disruption of gene(s) or parts of it has always been indispensable to understanding the function of the genes or gene segments^15^,^16^,^17^. However, in bacteria, rational mutagenesis of essential genes, often involved in fundamental biological process, is seldom accomplished in an efficient manner directly on the chromosome. In most cases, a non-native setting, such as episomally-encoded genes are required for generating such mutants and understanding gene-functions^17^,^18^,^19^. With the advent of the CRISPR/Cas9 system, chromosomal editing can be accomplished in an easier manner in prokaryotes but the application is limited to a small spectrum of bacteria due to the lack of native recombination machineries^8^,^9^,^10^,^11^,^12^,^13^,^14^.

In this study, we describe a unique genome-editing platform by combining the precise editing capability of the CRISPR/Cas9 system and the yeast homologous recombination machinery, and demonstrate robust and extensive chromosomal engineering of an essential bacterial gene that is conserved across all kingdoms, the *rrs* gene encoding the small-ribosomal subunit (16S) RNA. The ribosomal RNAs are particularly challenging to engineer, as opposed to other genes involved in replication, transcription or translation because of the presence of multiple copies of this gene in almost every genome, which adds another layer of complexity towards generating mutants^20^. These impediments to generating mutations in the small-ribosomal subunit RNA has made it difficult to dissect the functional role of different segments of this gene to understand various molecular mechanisms of translation. Conventionally, special *E. coli* strains lacking all the seven chromosomally-encoded rRNA operons and carrying a single, episomally-expressed rRNA operon have been used to engineer the 16S rRNA^21^,^22^. Apart from the requirement of generating and cloning the mutant 16S rRNA gene, this process also involves an arduous approach to replace the resident wild-type rRNA encoding plasmid with the mutant rRNA to obtain a pure population of the mutant ribosomes^20^,^21^. Further, this mutagenesis strategy can be even more complicated with functionally-debilitating phenotypes that induce recombination between the wild-type and the mutant plasmids or between the wild-type plasmid and the chromosome, resulting in mixed ribosomal populations, thus making it impossible to test the functional significance of such mutations. Alternative approaches to small-subunit rRNA gene engineering involves site-directed mutagenesis methods used in *Mycobacterium smegmatis*^23^,^24^. In this approach, the wildtype allele is replaced by the one carrying mutations by using RecA-mediated homologous recombination *in vivo*^23^,^24^. However, the feasibility of this method is limited by the ability to select for the mutations with a direct phenotype (gentamycin-resistance, in this case) or for another *cis*-mutation with a phenotype such as antibiotic resistance that accompanies the desired 16S rRNA mutation, since the RecA-mediated homologous recombination is not efficient enough to allow marker-free selection of recombinants. For example, the mutant 16S rRNA gene is co-selected by a mutation in the large-subunit RNA, A2058G (*E. coli* numbering), that renders cells resistant to the antibiotic clarithromycin, hence enabling the cells with the 16S rRNA mutation to be selected on clarithromycin^23^,^24^. Thus, through this method, it is impossible to engineer and study pure mutants without a selection-phenotype.

We chose the *Mycoplasma mycoides* genome to demonstrate a new strategy to engineer the 16S rRNA gene because of the low copy number of the ribosomal operons in this genome but more importantly, because of the notable synthetic biology accomplishments completed with this genome including the chemical synthesis of the entire genome, cloning of this entire genome in yeast and rebooting of this genome using genome-transplantation^25^,^26^,^27^. We used the CRISPR/Cas9 system to engineer the 16S rRNA gene from the *Mycoplasma mycoides* genome cloned in yeast in a seamless and a marker-free manner. Moreover, we eliminated the commonly-used two-plasmid CRISPR/Cas9 expression system^7^,^28^ by using *in vitro* transcribed guide-RNAs and chromosomally-encoded Cas9, thereby simplifying this tool significantly without compromising its genome-editing efficiency.

## Results

### *In vivo* editing of the *Mycoplasma mycoides* genome using CRISPR/Cas9

In order to edit the genome of *Mycoplasma mycoides*, we cloned it as a circular yeast artificial chromosome (YAC)^27^ in the *Saccharomyces cerevisiae* str. VL6-48N-Cas9 (see methods), which constitutively expressed Cas9. For targeting a specific site within this *M. mycoides* chromosome, we transformed this yeast strain simultaneously with *in vitro* transcribed guide-RNAs (gRNA) and donor DNA molecules. Despite the obvious shorter-life of the transformed gRNA transcript in dividing yeast, compared to the gRNA expressed from a plasmid or from a PCR product^7^, this co-transformation method was surprisingly sufficient to obtain up to a 100% editing efficiency, in the absence of any selection for the donor molecule or the editing event. Importantly, we were able to use two gRNA transcripts simultaneously to target both ends of the *rrs* gene and replace it with the engineered synthetic rrs^*^ donor molecule that was usually a PCR product amplified from a pre-cloned plasmid. Since this was a marker-free chromosomal editing, we selected for the transformed yeast cells by including an “empty” plasmid along with the gRNAs and the donor PCR amplicon in the transformation mix. The “empty” plasmid is an “yeast artificial chromosome” vector that carries replication elements necessary for maintenance in *Saccharomyces cerevisiae* and also carries a TRP1 marker to enable selection on plates lacking tryptophan. Remarkably, even in the absence of the direct selection of the editing event, a majority of the cells that received the “empty” vector (selected on plates lacking tryptophan), had been edited at the *rrs* locus in the YAC (Supplementary Fig. 1 and Supplementary Table 1). Interestingly, the CRISPR/Cas9 mediated engineering of the *M. mycoides* genome, in most cases, did not result in any rearrangements within this YAC, as verified by multiplex-PCR (Supplementary Fig. 2) and subsequent genome-transplantation. Importantly, using this method, we were able to test the function of multiple donors (up to twenty-seven) simultaneously by transforming a pool of the donor molecules along with the gRNAs, demonstrating the power of selection in this system.

The wild-type *M. mycoides* genome carries two ribosomal RNA operons. However, we recently synthesized a minimal cell, JCVI-Syn3.0, which was designed based on the gene content of *M. mycoides*^29^. In the process of designing the JCVI-Syn3.0 genome, we discovered that either of the two ribosomal operons was sufficient to support cellular viability. Hence, we used a version of the *M. mycoides* genome carrying only one of the ribosomal operons, which facilitated 16S rRNA engineering and testing functionality.

**Figure 1:**
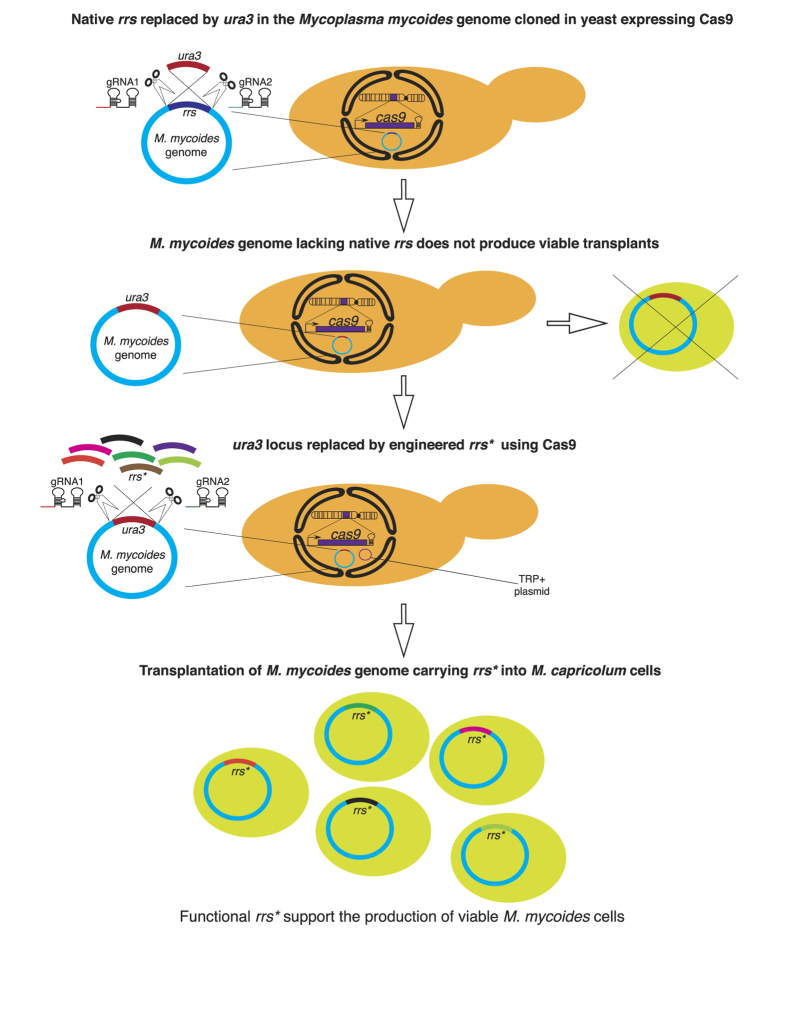
Overview of the 16S rRNA engineering process using CRISPR/Cas9. Workflow used to generate *M. mycoides* genome carrying engineered small-subunit RNA gene, rrs*.VL6-48N strain constitutively expressing Cas9 was used to maintain the *M. mycoides* genome as a circular yeast artificial chromosome. This strain was electroporated with two *in vitro* transcribed gRNAs and a donor *ura3* cassette to replace the wild-type *rrs* gene in *rrnII* operon, resulting in a *ura* + strain and a *M. mycoides::*Δ*rrs* genome that did not produce any functional *M. mycoides* cells upon genome transplantation into the recipient *M. capricolum* cells. *ura3* cassette in the *M. mycoides::*Δ*rrs* genome was subsequently replaced by engineered rrs* or wild-type *rrs* by using a single donor or a pool of rrs* donors along with two *in vitro* transcribed gRNAs and the “empty” pYAC_TRP1 plasmid. Transformants were screened for the *rrs* replacement of the *ura3* cassette in the genome and the integrity of the *M. mycoides* genome using multiplex-PCRs. At least three yeast positive clones were chosen for genome transplantation and testing cellular-viability if a single donor *rrs* was used. Transformants from entire plates (>1000 c.f.u) were scrapped, co-cultured and used for genome transplantation when pools of rrs* were used as donors during transformation.

As a first step towards introducing the engineered rrs* in the *M. mycoides* genome, we replaced the existing copy of *rrs* with the yeast *ura3* auxotrophic marker using CRISPR/Cas9 (Fig. 1). As expected, the resulting genome, without any *rrs* gene, was non-functional and did not produce any viable *M. mycoides* cells upon genome transplantation. This enabled an efficient functional-selection system, where the newly designed rrs* genes could be added to replace the *ura3* cassette and only functional rrs* constructs were able to restore the capacity of the genome to support viability (Fig. 1). We tested this hypothesis by using the wild-type *M. mycoides rrs* as a control to replace the *ura3* gene and observed cellular viability upon genome transplantation (Supplementary Fig. 1).

### Replacing the entire *M. mycoides rrs* gene with evolutionarily distant heterologous *rrs* did not support viability

**Figure 2:**
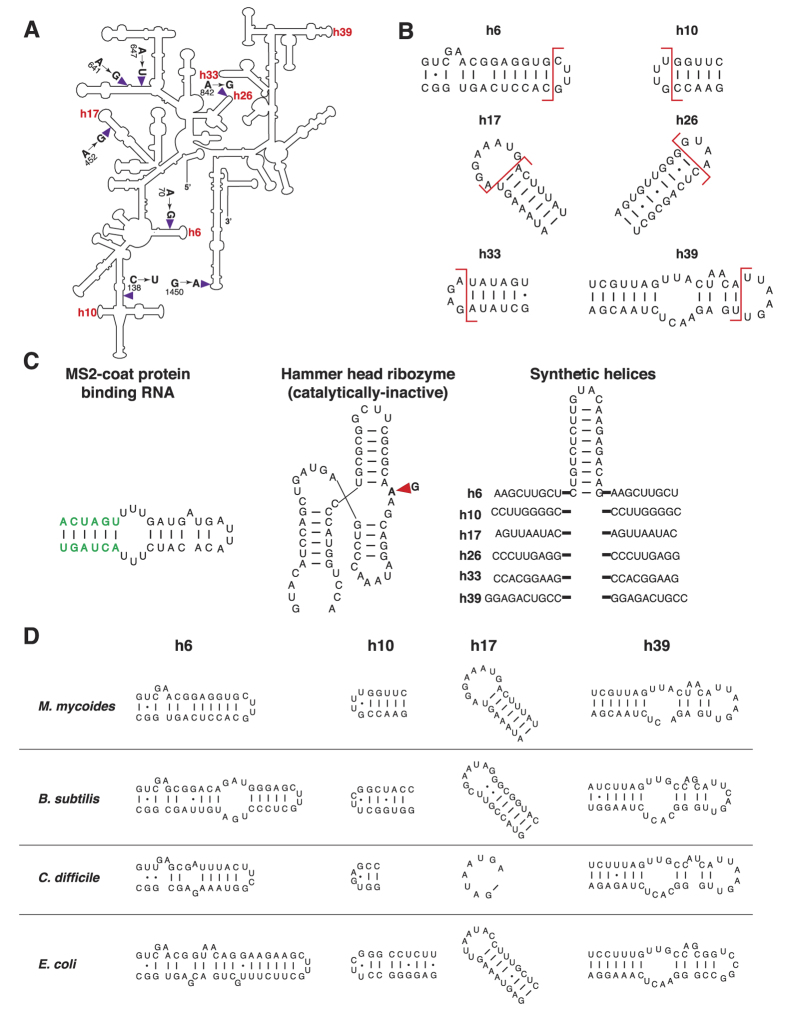
Sites and sequences used in *rrs* engineering. **(A)** Secondary structure of the *M. mycoides* 16S rRNA in which the six locations where the gene was engineered and the seven mutations incorporated from the *M. capricolum rrs* are indicated. *rrs* carrying *M. capricolum* mutations was used as a template to generate the single-, dual- or triple-site mutations. **(B)** Sites where of insertion elements were added to the *M. mycoides* 16S rRNA. Positions at which each of the six helices were “opened” to insert heterologous elements are shown using red lines. **(C)** Insertion elements used in *rrs* engineering. Sequence of the MS2-coat protein binding RNA (MS2), the catalytically-inactive hammer-head ribozyme (HHRi) and the synthetic, scar helices (SH) are shown. Sequences of the MS2 and HHRi elements added to each of the six sites shown in **(A)** are the same while the SH element added to each one of six sites varied in the sequences flanking the common hairpin structure. MS2 element was flanked by SpeI sites (green) as described in Youngman *et al*.^34^. G→A mutation that renders the hammer-head ribozyme catalytically-inactive is indicated. **(D)** Helix substitutions. Four helices, h6, h10, h17 and h39 from *M. mycoides* were substituted with those from *B. subtilis, C. difficile* and *E. coli*. Sequences and secondary structures of the wild-type and the heterologous helices that were used for substitutions are shown.

Even though as little as a single point mutation within the *rrs* could be lethal^30^, the functionally-significant parts of this gene are heavily conserved across all three kingdoms of life. Hence, as a first attempt at understanding the extent to which the *rrs* could be engineered, we replaced the entire gene (within the “mature” rRNA coding region of the gene) with that from a variety of phylogenetically distant bacteria such as *E. coli, B. subtilis* and *Clostridium acetobutylicum, Chloroflexus aggregans, Deinococcus radiodurans, Streptomyces scabiei, M. capricolum, Mesoplasma florum, Spiroplasma taiwanese* and *Acholeplasma laidlawii* (sequences listed in Supplementary information). Not surprisingly, transplants were obtained only when the *M. capricolum rrs* was substituted for that of *M. mycoides* because *M. capricolum* carries the phylogenetically closest *rrs* gene to *M. mycoides* with only seven nucleotide substitutions (Fig. 2A and Supplementary Fig. 3).

### Rational engineering of the *M. mycoides rrs* gene

Since the entire *rrs* gene could not be extensively engineered at the same time by substitution of the whole coding region, we rationally identified regions where helix substitutions or non-native elements could be introduced. We chose six sites within the *rrs* gene (Fig. 2B), helices 6, 10, 17, 26, 33 and 39, due to the following reasons: a) based on the crystal structures^31^ and rRNA modification studies^32^, these six helices do not associate with ribosomal proteins, so that idiosyncratic associations, if any, between the *M. mycoides* proteins and the rRNA would not perturbed if these sites are engineered (Supplementary Fig. 4), b) these solvent-exposed helices are also the places where expansion segments have evolved in higher eukaryotes^33^, indicating that they could potentially be more amenable for engineering, and c) previous engineering efforts have been successfully attempted at these helices in *E. coli*, which resulted in functional ribosomes^33^,^34^,^35^,^36^.

For insertion of heterologous elements in the *M. mycoides rrs*, we chose the previously established genetic insertions in the *E. coli* rRNA such as a) the MS2 viral coat-protein binding sequence (MS2)^34^, b) the hammerhead ribozyme (catalytically-inactive version) (HHRi)^35^, and c) the synthetic “scar helices” (SH), which were remnants from Tn5 transposon events on the *E. coli* rRNA^33^ (Fig. 2C). In addition to the insertion of heterologous structures, we also attempted to replace entire helices with those from phylogenetically -related (*B. subtilis* and *C. difficile*, which belong to the same phylum as *M. mycoides*, Firmicutes) or -distant bacteria (*E. coli* which belongs to the phylum Proteobacteria) (Fig. 2D). Since the *M. capricolum rrs* could support the growth of *M. mycoides*, we used this gene as a chassis to further engineer the *M. mycoides* 16S rRNA with addition of genetic elements or replacement of helices at specific locations. We introduced the three heterologous elements described above as insertions at all six of the selected locations, namely, helices 6, 10, 17, 26, 33 and 39 (Fig. 2b,C), but we created helix substitutions only at four helices, namely, 6, 10, 17 and 39 (Fig. 2D), because while mutations at helices 26 and 33 support cellular viability, they also slow down the growth rate^33^.

### Addition of heterologous genetic elements were more tolerated than helix substitutions

We designed and synthesized 18 novel rrs* genes carrying insertions of MS2, HHRi or SH at six locations: h6, h10, h17, h26, h33 and h39 and twelve more rrs* carrying helical substitutions at h6, h10, h17 and h39 from *E. coli, B. subtilis* or *C. difficile*. In general, the addition of genetic elements mostly preserved the sequence and architecture of the native helices since they were added as an extension of the existing helices (Fig. 2B), while the helix substitutions did not.

**Figure 3:**
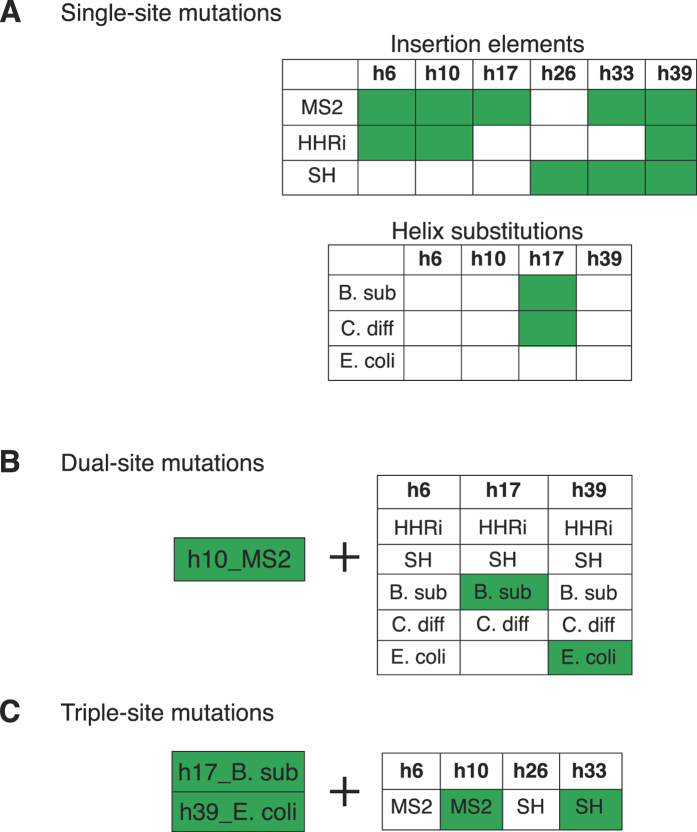
Results of the *M. mycoides* 16S rRNA engineering. **(A)** Single-site engineering. For the 16S rRNA carrying additional insertion elements, 11/18 rrs* produced viable transplants while 2/12 rrs* carrying helices substituted with those from different bacteria produced viable transplants. MS2 added to five helices (h6, h10, h17, h33 and h39), HHRi added to three helices (h6, h10, h39), SH added to three helices (h26, h33, h39) and h17 substituted with that from *B. subtilis* or *C. difficile* produced viable *M. mycoides* cells. **(B)** Dual-site engineering. Fourteen variations were added to the h10_MS2 rrs* at h6, h17 and h39, out of which two yielded viable *M. mycoides* cells upon genome transplantation. Two rrs* dual-site positives carried h17 or h39 substitutions from *B. subtilis* and *E. coli*, respectively, together with the h10_MS2 insertion element. **(C)** Triple-site engineering. To the dual-site variant carrying h17 and h39 substitutions from *B. subtilis* and *E. coli*, respectively, MS2 or SH insertion elements were added at h6 and h10 or h26 and h33, respectively, to create four rrs* triple-site variants. Out of these two rrs* produced functional transplants, with insertions h10_MS2 or h33_SH along with the two helix substitutions.

The newly engineered rrs* genes were introduced into the *M. mycoides* genome using CRISPR/Cas9 and whole-genome transplantation was performed into the recipient *M. capricolum* cells. Interestingly, we found that eleven out of the eighteen (>60%) rrs* carrying additional genetic elements produced viable transplants while only two out of the twelve rrs* with heterologous helix replacements produced viable transplants (Fig. 3A). The addition of the MS2 sequence seemed to be the most benign among the insertions, producing transplants in five out of six helix locations tested, with the exception of h26 (Fig. 3A). The other two insertion elements, HHRi and SH, gave viable *M. mycoides* transplants when added at three helix locations. Interestingly, the only one out of three locations coincided for the two insertion elements in terms of producing transplants, namely h39. Apart from h39, HHRi inserted at h6 and h10 produced transplants while SH added at h26 and h33 resulted in viable *M. mycoides* cells (Fig. 3A). Curiously, among the helix replacements, the two constructs for which transplants were obtained had heterologous h17, wherein the *M. mycoides* sequence was replaced with helices from *B. subtilis* and *C. difficile* (Figs 2D and 3A).

### Engineering multiple sites simultaneously demonstrates plasticity of the *M. mycoides rrs*

With many of the single-site engineering designs producing viable *M. mycoides* cells, we built on this success and incorporated mutations at two locations simultaneously within the same *rrs* molecule. We tested a handful number of combinations of the single-site mutations by first choosing the MS2 insertion at the h10 position as our base construct because the MS2 insertion seemed to be the least intrusive structure to *rrs* function in supporting viability (Fig. 3A). Also, among the four helix positions (h6, h10, h33, h39) that exhibited viability with at least two different heterologous insertions, we chose h10 randomly. For designing the dual-site mutations, we chose to engineer h6, h17 and h39, by adding genetic elements and substituting helices together with the MS2 insertion at h10 (Fig. 3B). Even though we obtained transplants with rrs* carrying MS2 (h33) and SH (h26 and h33) insertions, we did not use the known problematic sites (h26 and h33)^33^ for the dual-site engineering. Among the fifteen constructs designed to carry mutations simultaneously at two helices, we were able to clone and test fourteen of them, with the exception of the construct carrying h17 from *E. coli* and MS2 addition at h10. Among the fourteen constructs tested, we obtained transplants for two constructs, both of which carried helix substitutions either at h17 from *B. subtilis* or h39 from *E. coli* in addition to the MS2 insertion at h10 (Fig. 3B).

We advanced the multi-site engineering of the *rrs* even further by designing constructs carrying mutations at three locations simultaneously. Based on our previous success with the single- and dual-site variants, we carefully designed four *rrs* constructs that carried three engineered sites (Fig. 3C). The experiments with the dual-variants clearly showed that the *M. mycoides rrs* is unable to tolerate two different heterologous insertions when introduced simultaneously at two distinct locations. Hence, for all the triple-site engineered constructs, we used the rrs* carrying two helix substitutions that produced viable transplants when used as a part of the dual-site variants (Fig. 3B), namely, h17 from *B. subtilis* and h39 from *E. coli*. To this construct, a single insertion element such as the MS2 element at h6 or h10 or the SH element at h26 or h33, was added (Fig. 3C). The HHRi element was not used because it is the largest addition to the *rrs* gene that we have tested (60 nucleotides) (Fig. 2C), hence, we hypothesized that it would be highly unlikely to obtain viable *M. mycoides* cells if it is used in conjunction with two other mutations for the triple-site engineering. Among the four triple-site mutant *rrs* constructs tested, we obtained viable transplants for two of them carrying MS2 or SH insertion elements at h10 or h33, respectively, alongside the h17 and h39 substitutions (Fig. 3C).

## Discussion

The use of CRISPR/Cas9 for genome editing has had a profound impact in the field of synthetic biology and more importantly, continues to open new avenues for translational medicine such as gene-therapies^37^. In this study, we utilized the potential of CRISPR/Cas9 and the innate homologous recombination capacity of yeast to engineer the small-subunit ribosomal RNA in the bacterial genome of *M. mycoides*. Furthermore, we also tested the function of the newly-introduced mutations by transplanting the genome with the engineered 16S rRNA gene into recipient *M. capricolum* cells^27^. Thus, we were able to generate a genome-engineering platform that can be used for robust and extensive site-directed mutagenesis directly on the bacterial chromosome with up to 100% efficiency.

The ribosomal RNAs are one of the most conserved genes across all three kingdoms of life, underscored by the fact that the small-subunit RNA is used to measure the rate of evolution of organisms and phylogenetically-classify them according to the divergence in the sequence of this gene. Apart from the conserved-nature, engineering the 16S rRNA presents a considerable challenge also because of the number of copies of the ribosomal RNA genes varies between 1 and 15 within the domain *Bacteria*^20^. Functional redundancy of various copies of the rRNA has been demonstrated in few eubacterial species such as *E. coli*^21^,^22^ and *M. smegmatis*^23^,^24^ as well as in the eukaryote, *Saccharomyces cerevisiae*^38^ which has enabled the engineering of organisms with homogenous populations of ribosomes encoded by single transcription units. Thus the copy-number problem was circumvented by using model organisms such as *M. smegmatis*, which only has two copies of rRNA genes^23^,^24^ and *E. coli* strains with a single rRNA operon encoded from a plasmid or the chromosome^21^,^22^. Likewise, the *M. mycoides* genome carries only two rRNA operons, and importantly, deletion of one operon does not affect viability of the cells (Hutchison *et al*.^29^). Hence, this makes *M. mycoides* an ideal model for engineering the 16S rRNA and the ribosome. However, the lack of genetic tools has limited the use of *M. mycoides* as a model organism to study fundamental biological processes despite the many ground-breaking synthetic biology-feats accomplished with its genome^25^,^26^,^27^. One of these accomplishments, namely, cloning of the entire bacterial genome in yeast, allowed us to apply the genetic tools developed in yeast on the *M. mycoides* genome, thus making it a versatile platform for bacterial genome editing. Using the unique capacity of this platform, we were able to generate a version of the genome lacking the essential 16S rRNA gene, which is not possible by conventional techniques. Subsequently, the functionality of this non-functional genome was restored by using wild-type or an engineered version of the gene that could support ribosome function. Additionally, we were able to test the function of multiple versions of the engineered 16S rRNA gene in a single yeast-transformation and the subsequent *M. mycoides* genome transplantation event (Supplementary table 1), thus creating the possibility of high-throughput functional-testing of multiple loci through this platform.

The goal of this study was not to systematically understand if every segment of the *M. mycoides rrs* gene is essential for its function. Rather, our study aimed at using the *M. mycoides* genome cloned in yeast as a platform to explore the possibilities of engineering this essential gene by identifying the sites where sequence modifications could be introduced and the extent to which such modifications could be tolerated. In the process, we found that the introduction of the MS2 coat-protein binding helix (MS2) was the most tolerated heterologous element since five out of six engineered rrs* carrying this modification at different locations did not affect cellular viability. Notably, in *E. coli*, the introduction of MS2 at h6 did not affect viability; however, it only resulted in ~85% of the MS2-tagged small-subunits upon affinity purification^34^. Given our results with MS2, perhaps the introduction of MS2 at multiple sites simultaneously could have still allowed for fully-functional ribosomes and cellular-viability in *E. coli* and recovered a much higher fraction of the MS2-tagged subunits. Similarly, the introduction of the MS2 element at multiple locations in the large-subunit rRNA could have resulted in a purer population than the observed ~90% when tagged at a single site^34^. Also of note, the problem of heterogeneity for non-lethal mutations is circumvented in the *M. mycoides* platform, since the wild-type gene is completely eliminated before the introduction of mutagenized 16S rRNA gene.

It is interesting to note that all six sites chosen for engineering (h6-h39) were able to tolerate at least a single kind of an insertion element (Fig. 3A), indicating that for the rrs* carrying insertions that did not produce viable transplants, the site was not the reason *per se*. However, the context of the insertion elements could have played a role in the rRNA folding following transcription, post-transcriptional modifications, association of ribosomal proteins or rRNA stability, thus affecting the small-subunit assembly and function and consequently, cellular viability. For example, the insertion of “synthetic helices” has been shown to modestly decrease the fidelity of the ribosome (less than two-fold) in-terms of stop-codon read through and frameshifts^33^. However, a detailed functional knowledge of how each of these six helices contribute to ribosome function is largely unknown. The structural integrity and hence, the functionality of the native helices are largely preserved during the addition of heterologous elements, while helix substitutions deviate considerably in sequence and consequently, in the structure of the helix (Fig. 2B,D). This could potentially explain why most of whole helix substitutions were lethal; only 2/12 single helix substitutions produced viable transplants as opposed 11/18 helix insertions. On the contrary, we observed a loss of viability when two distinct insertion elements were introduced at two different locations simultaneously (Fig. 3B), even though we observed viability when both h17 and h39 were substituted simultaneously with those from *B. subtilis* and *E. coli*, respectively (the triple-site engineered rrs* that were functional carried these two substitutions, Fig. 3C).

While the insertion elements were already tried and tested in *E. coli*^33^,^34^,^35^,^36^, it was still surprising to note that several of these single-site additions were able to support viability, given the considerable 16S rRNA phylogenetic distance between *E. coli* and *M. mycoides* (Supplementary Fig. 3). Even more impressively, we were able to generate functional rrs* engineered at up to three locations (Fig. 3c) within the gene by careful design, which clearly demonstrates the flexibility provided by the architecture of the 16S rRNA. This flexibility could be exploited to add new functions to the translation machinery such as orthogonality and also to study central ribosome functions such as initiation and decoding by introducing mutations. Also of note, all the single-, dual- and triple-site variants were tested on the *M. mycoides rrs* that already carried seven mutations from the *M. capricolum rrs* gene. It is likely that perhaps even more of these engineered rrs* could have been functional had they been tested in the context of the wild-type *M. mycoides rrs* gene, underscoring the potential utility of this platform to generate and study small-subunit mutants.

Our efforts were focused mainly on evaluating if the engineered rrs* could support the production of viable *M. mycoides* cells after genome transplantation. An easily observable phenotype of rRNA engineering is the growth-defect brought about by the mutations introduced. It is conceivable that some of the transplants carrying engineered rrs* are slow-growing upon culturing, which might even explain in some cases, why mutations that produced transplants individually did not produce viable *M. mycoides* cells when combined (Fig. 3A–C). Such slow-growing mutants could be potentially used to study processes such as 16S rRNA folding and stability and the small-subunit maturation.

In conclusion, the *M. mycoides* platform described here could enable the high-throughput testing of different mutagenized versions of any essential gene since only the functional versions can restore the capacity of the genome to produce viable cells upon genome transplantation. Thus, this platform can be applied to further understand the function of various regions of the 16S rRNA and could be extended to the large-subunit RNA, ribosomal and extra-ribosomal factors and other essential genes involved in fundamental biological processes to answer basic questions of life.

## Materials and Methods

### *Mycoplasma mycoides* genome

*M. mycoides* genome used in this study carried a yeast CEN/ARS and a HIS3 marker for centromeric propagation of the genome in yeast. The *rrnI* operon was initially deleted from the *M. mycoides* genome and the *rrs* gene in the operon *rrnII* was engineered. A partially-minimized version of the genome was used in this study. Specifically, 1/8^th^ of the genome, comprising the *rrnI* operon, was minimized, which did not affect the growth rate of the organism, was used^29^.

### Construction of Cas9 expressing yeast strain

The *cas9* gene from *Streptococcus pyogenes* was originally codon-optimized and constructed for expression in human cells. This construct was ordered and obtained from DNA2.0. The *cas9* gene was both N-terminally and C-terminally tagged with a nuclear localization signal. The plasmid p416TEF1 was cut with BamHI and XhoI, and the vector backbone with the TEF promoter and CYCt1 terminator was gel purified. The *cas9* gene was PCR amplified from the original construct provided by DNA2.0 and assembled into the linearized p416TEF1 vector using the Gibson HiFi^TM^ Assembly^39^ kit (SGI-DNA). The assembly reaction was transformed into electrocompetent *E. coli* cells, TransforMax^TM^EPI300^TM^ (Illumina), and positive clones were identified.

The *Saccharomyces cerevisiae* strain used in this study was VL6-48N (ATCC #MYA-3666). These cells were transformed with 100 ng of p416TEF-cas9, 1 μg of sgRNA and 1 μg of the PCR-amplified TEF1p-cas9-cyc1t expression cassette (4.9 kb). Cells were plated on selection plates lacking uracil. Integration of the tef1p-cas9-cyc1t cassette at the chromosome locus YAL044W-A was verified by diagnostic colony PCR and the positive clones were patched on 5-FOA selection plates to eliminate the p416TEF-cas9 plasmid. The integrated cas9 cassette was sequenced and the analysis revealed a point mutation at amino acid position 871, with histidine replacing the proline at this position. Surprisingly, the clone carrying the amino acid substitution was functional and hence, this yeast strain was used for *M. mycoides* genome engineering.

### sgRNA production

sgRNA was produced by *in vitro* transcription with the MEGAshortscript T7 transcription kit (Life Technologies). Two complimentary DNA ultramers were designed to carry a T7 promoter, 20-base spacer region, specific to the target, and the structural component of the sgRNA (Supplementary Table 3). The ultramers were ordered from IDT at 4 nmol scale and re-suspended in the annealing buffer (10 mM Tris-HCl pH 8.0, 50 mM NaCl, 1mM EDTA) to a final concentration of 50 μM. The ultramers were annealed by mixing equal volumes in 1.5 ml microfuge tube and heating up to 90–95 °C for 3–5 minutes in a heat-block. Following this incubation, the heat-block was allowed to cool to room temperature for 45–60 min. The annealed oligonucleotides were diluted to 1 μM with RNase-free water. 30 μl of the diluted ultramers were used as template in a 100 μl transcription reaction with the MEGAshortscript T7 kit according to the manufacturer’s instructions. The transcription reaction was incubated at 37 °C for 4 hours or overnight followed by addition of 1 μl of the Turbo DNase provided in the kit and incubation at 37 °C for another 15  min. The reaction volume was adjusted to 750 μl with RNase-free water and 1/10^th^ volume of 3M sodium acetate, followed by phenol: chloroform extraction. RNA was precipitated following this with two volumes of 100% ethanol and washed with 500 μl of 70% ethanol. The pellet was air-dried and then re-suspended in 100–200 μl RNase-free water.

### Transformation and screening for edited clones

Transformation was carried out *via* electroporation. Briefly, 50 ml of yeast culture was inoculated with 2ml of the overnight culture and incubated at 30 °C for 5 hours with vigorous shaking. The culture was harvested by centrifugation at 2000 *g* for 3 min. The pellet was washed twice with 50 ml cold, sterile distilled water and re-suspended in 800 μl of 100 mM lithium acetate solution in 1X TE buffer (pH-8). 20 μL of 1 M DTT was added and the cells were incubated at 30 °C for 45 min with gentle shaking. Cells were washed with 1 mL of cold sterile water, followed by a wash with 1 mL of ice-cold 1 M sorbitol solution and finally re-suspended in 500 μL of 1 M sorbitol solution. 100 μl of cells were used per electroporation with 1 μg of each sgRNA, 0.5–1 μg of single donor cassette or a pool of donor cassettes, and 100  ng of selection plasmid, pYAC_TRP1. Cells were electroporated under the following conditions: 2.5 kV, 200 Ohms, 25 μF. Electroporated cells were transferred into 1 ml of 1:1 mix of 1 M sorbitol/YEPD media, incubated at 30 °C overnight with shaking and plated on selection plates without tryptophan and histidine. Plates were incubated at 30 °C for 2 days and several transformants were patched on to selection plates without histidine before screening for the chromosome editing event and the integrity of the *M. mycoides* genome. Screening was done using multiplex PCRs with the QIAGEN multiplex PCR kit according to the manufacturer’s instructions (diagnostic PCR primers are listed in Supplementary Table 4 and 5). Positive yeast clones were used to transplant the *M. mycoides* genome into the *M. capricolum* cells as described in Lartigue, C. *et al*.^25^. The resulting transplants were screened by amplifying and sequencing the resident *rrs* gene. Some clones were further verified for the lack of wild-type copy of the *rrs* gene by performing whole-genome sequencing and *de novo* assembly.

### Preparation of donor DNA for *rrs* engineering

The donor cassettes carrying *rrs* from *E. coli, B. subtilis* and *Clostridium acetobutylicum, Chloroflexus aggregans, Deinococcus radiodurans, Streptomyces scabiei, M. capricolum, Mesoplasma florum, Spiroplasma taiwanese* and *Acholeplasma laidlawii* were synthesized from oligonucleotides ordered from IDT using the procedure described in the patent US2014-0308710 and the sequences are reported in Supplementary information. *M. capricolum rrs* was used to generate single-, double- and triple-site mutants. The variations such as helix insertions and substitutions in the *rrs* gene were introduced during PCR and producing overlapping PCR products. Primer sequences are mentioned in Supplementary Table 2. PCR fragments were assembled and cloned into pSGI-Bac01 vector using the Gibson HiFi^TM^ Assembly kit (SGI-DNA) according to the manufacturer’s instructions and sequence-verified (SGI-DNA). Donors were PCR-amplified from sequence-verified clones using Q5^®^ High-Fidelity 2X Master Mix (New England Biolabs) and used for Cas9-mediated *rrs* engineering.

### Genome transplantation

Previously published genome transplantation protocols were utilized for testing the viability of the engineered *M. mycoides* genomes^26^,^27^,^28^,^29^. Briefly, yeast colonies carrying *M. mycoides* genome were grown up to an OD_600_ of 1.5. DNA from the yeast cells were captured on agarose plugs by mixing yeast cells with melted agarose and subsequent treatment of the plugs with Lyticase and Proteinase K enzymes. After inactivating Proteinase K with PMSF, yeast plugs were further washed with 1X TE (pH-8) solution and used for genome transplantation. Recipient *M. capricolum* cells were prepared by washing an exponential culture (~pH-6.2) with T/N buffer (10 mM Tris-HCl – pH – 7.5 and 250 mM sodium chloride) and resuspending the cells with 0.1M calcium chloride solution. For genome transplantation, the agarose plugs carrying the *M. mycoides* genomes were melted by β-agarase treatment and mixed with the recipient cells in the presence of PEG-6000. This mixture was incubated for 90 min at 30 °C, resuspended in SP4 growth medium and plated on antibiotic-selection plates carrying tetracycline (4 μg/ml).

Genome transplantation protocol was used to assess the rRNA redundancy in the *M. mycoides* genome, the non-functionality of the genome when the remaining single copy of *rrs* was replaced with *ura3* and the functional rrs* variants that could support viability by replacing the *ura3* gene.

## Additional Information

**How to cite this article**: Kannan, K. *et al*. One step engineering of the small-subunit ribosomal RNA using CRISPR/Cas9. *Sci. Rep.* **6**, 30714; doi: 10.1038/srep30714 (2016).

## Supplementary Material

Supplementary Information
