# Supplementary material for: One step engineering of the small-subunit ribosomal RNA using CRISPR/Cas9
Source: Sci Rep. 2016 Aug 4;6:30714. doi: 10.1038/srep30714 (PMC4973257; doi:10.1038/srep30714)
Supplement: Supplementary Information [file srep30714-s1.pdf]

One step engineering of the small-subunit ribosomal RNA using CRISPR/Cas9

Krishna Kannan,[1] Billyana Tsvetanova,[1] Ray-Yuan Chuang,[2] Vladimir N. Noskov,[2] Nacyra Assad-Garcia,[2] Li Ma,[2] Clyde A. Hutchison III,[2] Hamilton O. Smith,[1,2] John I. Glass,[2] Charles E. Merryman,[2] J. Craig Venter,[1,2] Daniel G. Gibson,*[1,2]

Affiliations:

[1]Synthetic Genomics, Inc., La Jolla, CA 92037

[2]J. Craig Venter Institute, La Jolla, CA 92037

***Correspondence to: dgibson@jcvi.org**

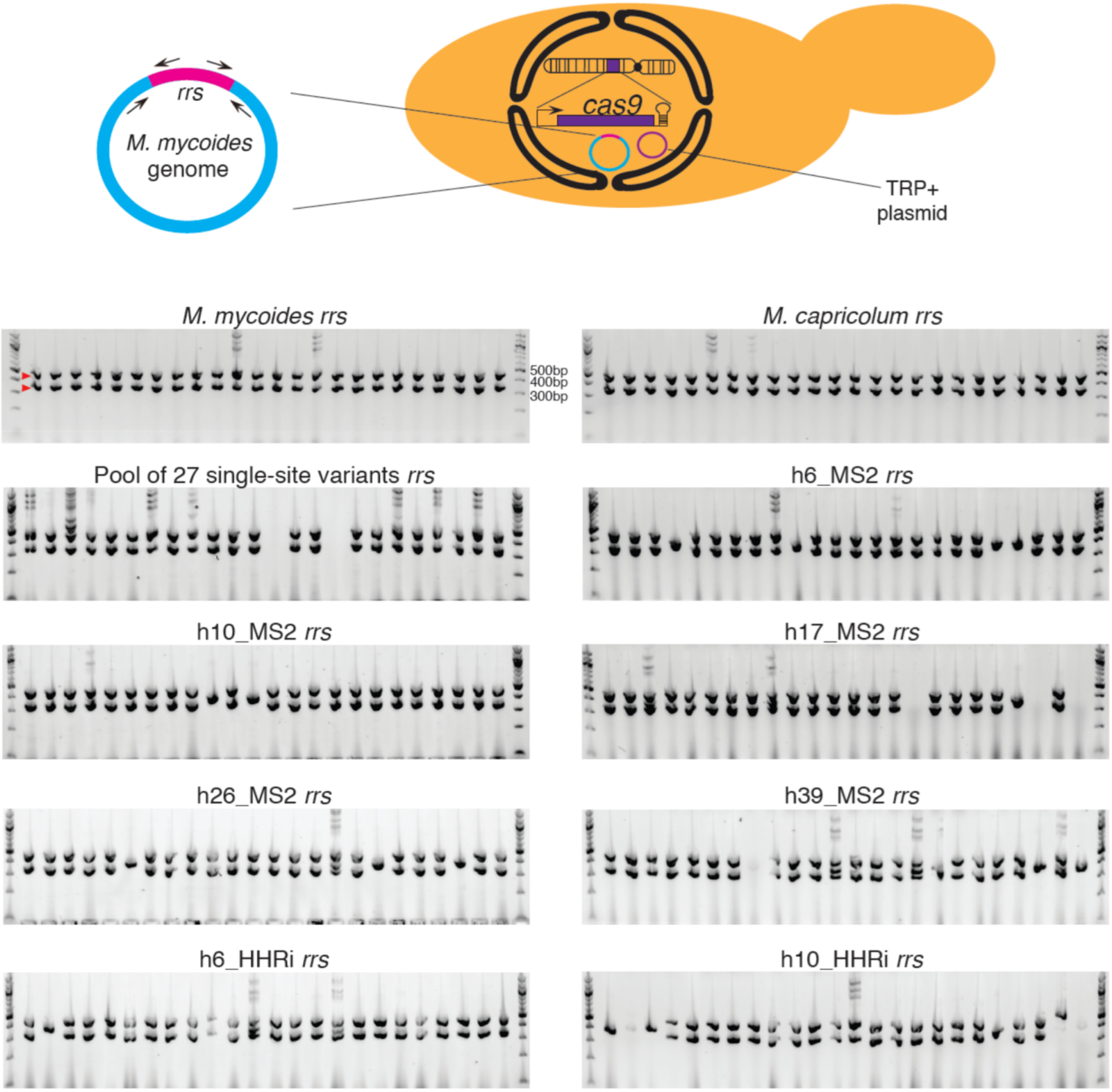

Supplementary figure 1. Detecting *M. mycoides* genome editing event using diagnostic multiplex PCR.

VL6-48N-Cas9 yeast strain carrying *M. mycoides Δrrs::ura3* genome was electroporated with two *in vitro* transcribed gRNAs (Supplementary Table 3), “empty” pYAC_TRP1 plasmid and donor *rrs* DNA. Transformants were selected on plates lacking histidine and tryptophan and screened by multiplex PCR (Primers listed in Supplementary Table 5) for the replacement of *ura3* cassette with *rrs* donor. Data from select donor transformations (*M. mycoides* and *M. capricolum rrs* and single-site variant donors used individually or as a pool) are shown after screening 24 transformants that were patched on to selection plate lacking histidine. The expected bands are indicated by red arrows (353bp and 474bp).

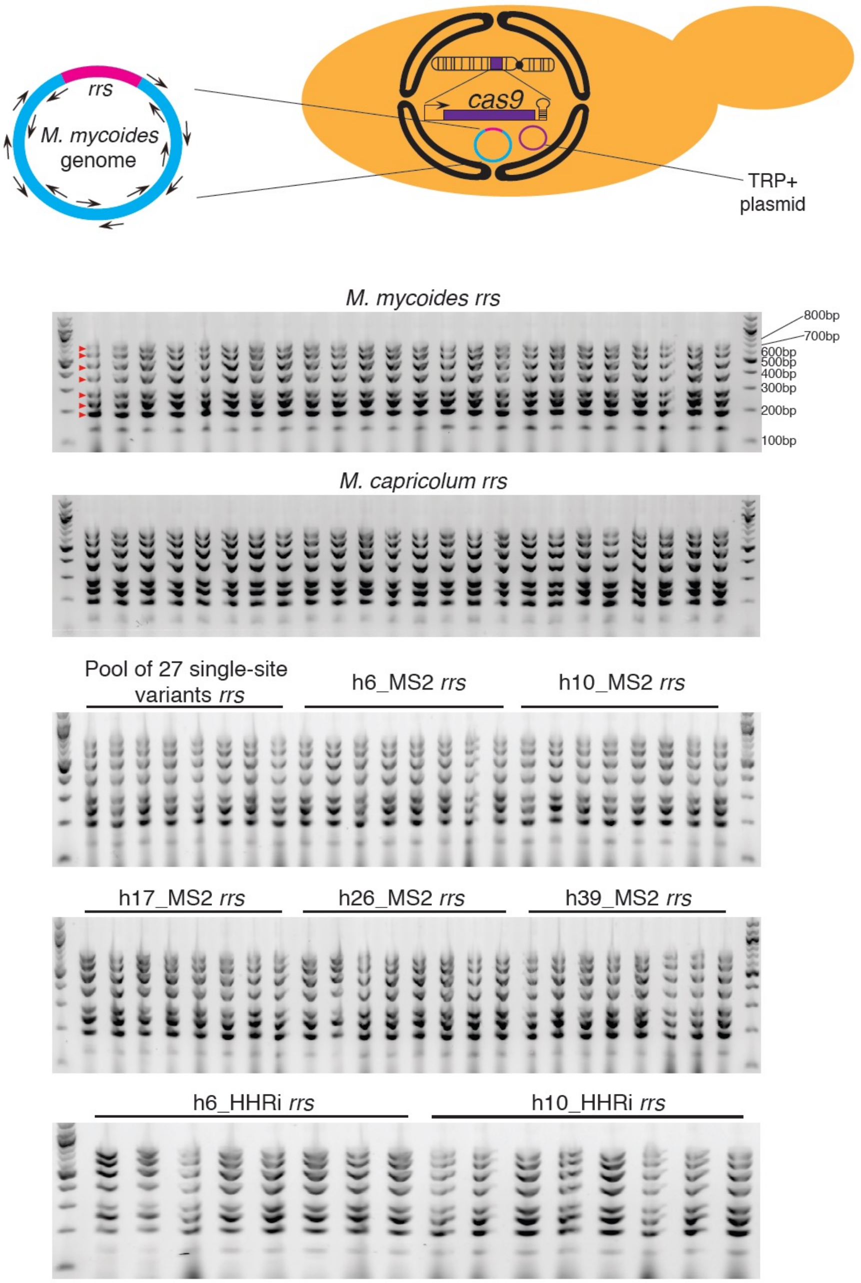

Supplementary figure 2. Testing the integrity of the edited *M. mycoides* genome using diagnostic multiplex PCR.

Twenty four (*M. mycoides* and *M. capricolum rrs* donors) or eight (single-site variants used individually or as a pool for donors) positive clones that were confirmed for *M. mycoides* genome editing at the *Δrrs::ura3* locus (Supplementary Fig. 1) were tested for rearrangements in within the YAC carrying the genome as a result of this editing using multi-locus multiplex PCR (Supplementary Table 4). Expected bands are indicated by red arrows.

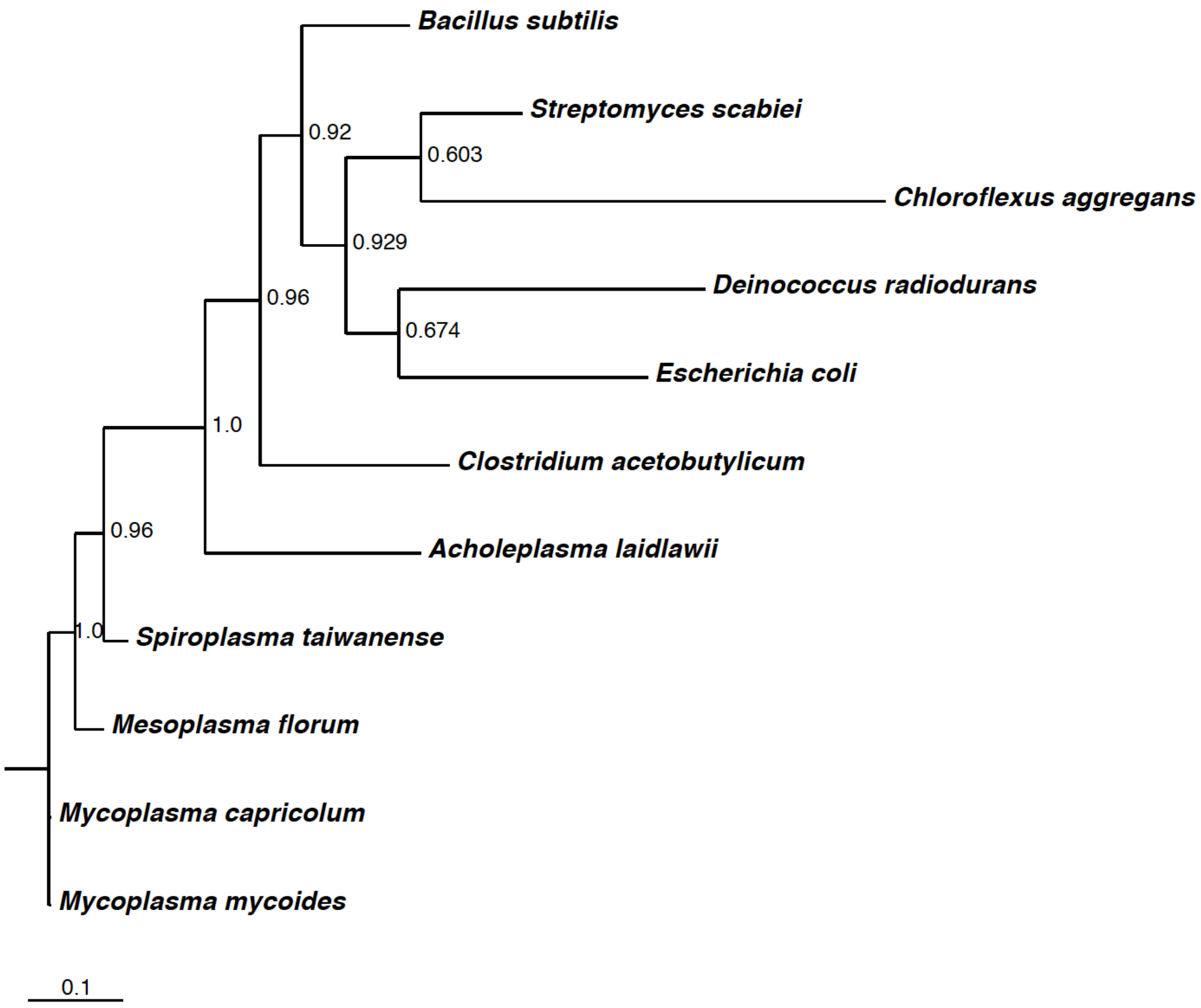

Supplementary figure 3. Phylogenetic analysis of heterologous *rrs* that were used to replace *M. mycoides rrs* and test viability.

In the *M. mycoides* genome carrying single rDNA operon, the *rrs* gene was substituted with *rrs* genes with variable phylogenetic-distances. Resulting genome was tested for viability by genome-transplantation and only *M. capricolum rrs*, carrying seven mutations compared to *M. mycoides rrs*, was able to support viability. Sequence alignment was performed using MUSCLE (http://www.ebi.ac.uk/Tools/msa/muscle/); phylogenetic analysis was performed using PHYML (http://phylogeny.lirmm.fr/phylo_cgi/phylogeny.cgi) and the tree was rendered using Phylodendron (http://iubio.bio.indiana.edu/treeapp/treeprint-sample1.html).

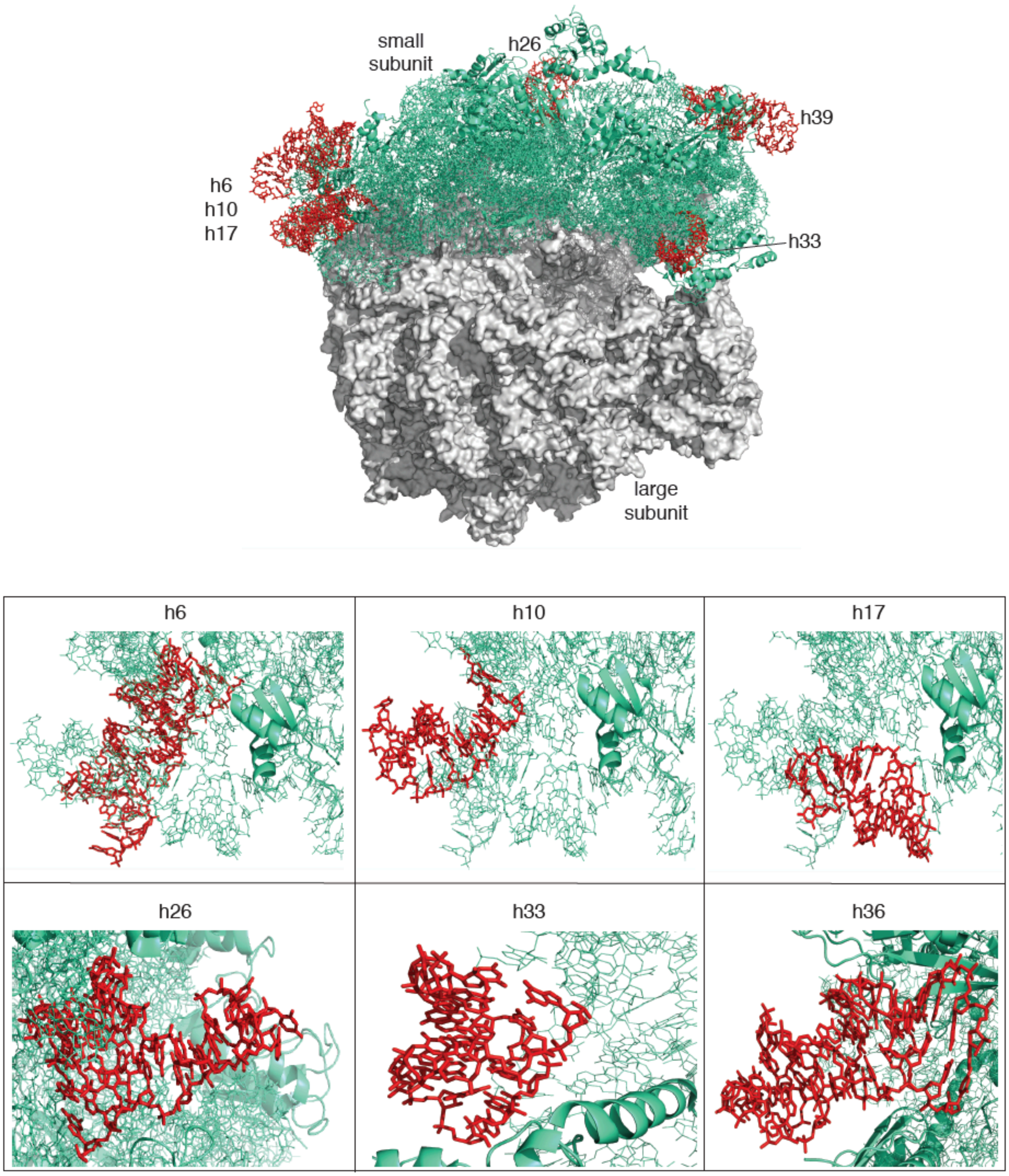

Supplementary figure 4. Location of helices 6, 10, 17, 26, 33 and 36 in *E. coli* ribosome.

Solvent-exposed helices 6, 10, 17, 26, 33 and 36 were chosen for engineering due of their lack of functional interactions with ribosomal proteins. Structure of the 70S ribosome with the large and the small subunits are shown (PDB IDs: 3OAR - 30S subunit and 3OAS – 50S subunit) and the locations of the six helices (red) on the 30S subunit (cyan) are highlighted.

Supplementary Table 1. *Mycoplasma mycoides* genome editing efficiency using CRISPR/Cas9.

Editing efficiency was calculated for select donor transformations (*M. mycoides* and *M. capricolum rrs*, single-site variant donors used individually or as a pool) from Supplementary Fig. 1. Twenty-four transformants from each transformation were screened.

Donor	Edited clones (%)
M. mycoides rrs	100
M. capricolum rrs	100
Pool (27 single-site variants)	92
h6_MS2	83
h10_MS2	92
h17_MS2	95
h26_MS2	87
h39_MS2	83
h6_HHRi	92
h10_HHRi	78

All 24 positive clones for *rrs* replacement when *M. mycoides* rrs or *M. capricolum rrs* was used as a donor, were tested for the integrity of the edited *M. mycoides* genome. For the other donors, eight positive clones were subsequently tested for genome integrity. None of these *rrs* positive clones showed any genome-rearrangements decipherable by multiplex PCR (Supplementary Fig. 2).

Supplementary Table 2. Sequences of primers used for *rrs* engineering.

Sequence	Name
AAGCTTGCTCTGTCTCTTGTACAAGAGACAGAAGCTTGCTCACCTCAGTG GCGAACGGGT	h6_SH_R
AGCAAGCTTCTGTCTCTTGTACAAGAGACAGAGCAAGCTTCACCCCCGTT CGACTTGCAT	h6_SH_F
CCTTGGGGCCTGTCTCTTGTACAAGAGACAGCCTTGGGGCGGTTCACTA TGAGATGGGGA	h10_SH_R
GCCCCAAGGCTGTCTCTTGTACAAGAGACAGGCCCCAAGGGGTTCTTTT GATCTTTTCTC	h10_SH_F
AGTTAATACCTGTCTCTTGTACAAGAGACAGAGTTAATACGACTTTATCTT GACAGTACC	h16_SH_R
GTATTAACTCTGTCTCTTGTACAAGAGACAGGTATTAACTACTCTATTTTT TCTTCCCTT	h16_SH_F
CCCTTGAGGCTGTCTCTTGTACAAGAGACAGCCCTTGAGGCTCAGCGCT GCAGCTAACGC	h26_SH_R
CCTCAAGGGCTGTCTCTTGTACAAGAGACAGCCTCAAGGGCCCAACACT TAGTACTCATC	h26_SH_F
CCACGGAAGCTGTCTCTTGTACAAGAGACAGCCACGGAAGTATAGTAGA GGTTAACATTG	h33_SH_R
CTTCCGTGGCTGTCTCTTGTACAAGAGACAGCTTCCGTGGTATAGCTTTG CACTGGATGT	h33_SH_F
GGAGACTGCCCTGTCTCTTGTACAAGAGACAGGGAGACTGCCTGAGAAC TCTAACGAGAC	h39_SH_R
GGCAGTCTCCCTGTCTCTTGTACAAGAGACAGGGCAGTCTCCTGTTAGT AACTAACGACA	h39_SH_F
TGGAATCCAGGACGCGCCGAAGCGCGTCCGTCCTATTTGGGACTCATCA GCTGGATGTACCACCCCCGTTCGACTTGCAT	h6_HHRi_F
GTACATCCAGCTGATGAGTCCCAAATAGGACGGACGCGCTTCGGCGCGT CCTGGATTCCACACCTCAGTGGCGAACGGGT	h6_HHRi_R
TGGAATCCAGGACGCGCCGAAGCGCGTCCGTCCTATTTGGGACTCATCA GCTGGATGTACGGTTCTTTTGATCTTTTCTC	h10_HHRi_F
GTACATCCAGCTGATGAGTCCCAAATAGGACGGACGCGCTTCGGCGCGT CCTGGATTCCAGGTTCACTATGAGATGGGGA	h10_HHRi_R
TGGAATCCAGGACGCGCCGAAGCGCGTCCGTCCTATTTGGGACTCATCA GCTGGATGTACACTCTATTTTTTCTTCCCTT	h17_HHRi_F
GTACATCCAGCTGATGAGTCCCAAATAGGACGGACGCGCTTCGGCGCGT CCTGGATTCCAGACTTTATCTTGACAGTACC	h17_HHRi_R
TGGAATCCAGGACGCGCCGAAGCGCGTCCGTCCTATTTGGGACTCATCA GCTGGATGTACCCCAACACTTAGTACTCATC	h26_HHRi_F
GTACATCCAGCTGATGAGTCCCAAATAGGACGGACGCGCTTCGGCGCGT CCTGGATTCCACTCAGCGCTGCAGCTAACGC	h26_HHRi_R
TGGAATCCAGGACGCGCCGAAGCGCGTCCGTCCTATTTGGGACTCATCA GCTGGATGTACTATAGCTTTGCACTGGATGT	h33_HHRi_F
GTACATCCAGCTGATGAGTCCCAAATAGGACGGACGCGCTTCGGCGCGT CCTGGATTCCATATAGTAGAGGTTAACATTG	h33_HHRi_R
TGGAATCCAGGACGCGCCGAAGCGCGTCCGTCCTATTTGGGACTCATCA GCTGGATGTACTGTTAGTAACTAACGACAAG	h39_HHRi_F

GTACATCCAGCTGATGAGTCCCAAATAGGACGGACGCGCTTCGGCGCGTCCTGGATTCCATGAGAACTCTAACGAGACTG	h39_HHRi_R
ACTAGTAAAGATGGGTAATCCTCATCAAAACTAGTCACCCCCGTTCGACTTGCAT	h6_MS2_F
ACTAGTTTTGATGAGGATTACCCATCTTTACTAGTCACCTCAGTGGCGAACGGGT	h6_MS2_R
ACTAGTAAAGATGGGTAATCCTCATCAAAACTAGTGGTTCTTTTGATCTTTTCTC	h10_MS2_F
ACTAGTTTTGATGAGGATTACCCATCTTTACTAGTGGTTCACTATGAGATGGGGA	h10_MS2_R
ACTAGTAAAGATGGGTAATCCTCATCAAAACTAGTACTCTATTTTTTCTTCCCTT	h17_MS2_F
ACTAGTTTTGATGAGGATTACCCATCTTTACTAGTGACTTTATCTTGACAGTACC	h17_MS2_R
ACTAGTAAAGATGGGTAATCCTCATCAAAACTAGTCCCAACACTTAGTACTCATC	h26_MS2_F
ACTAGTTTTGATGAGGATTACCCATCTTTACTAGTCTCAGCGCTGCAGCTAACGC	h26_MS2_R
ACTAGTAAAGATGGGTAATCCTCATCAAAACTAGTTATAGCTTTGCACTGGATGT	h33_MS2_F
ACTAGTTTTGATGAGGATTACCCATCTTTACTAGTTATAGTAGAGGTTAACATTG	h33_MS2_R
ACTAGTAAAGATGGGTAATCCTCATCAAAACTAGTTGTTAGTAACTAACGACAAG	h39_MS2_F
ACTAGTTTTGATGAGGATTACCCATCTTTACTAGTTGAGAACTCTAACGAGACTG	h39_MS2_R
GCCGCTAACATCAGGGAGCAAGCTCCCATCTGTCCGCTCGACTTGCATGTATTAGGCATG	h6_Bs_F
GTCGAGCGGACAGATGGGAGCTTGCTCCCTGATGTTAGCGGCGAACGGGTGAGTAACACG	h6_Bs_R
TTATCGCATGAGAAAAGATCAAAAGGTGGCTTCGGCTACCACTATGAGATGGGGATGCGG	h10_Bs_F
GGTGGCTTCGGCTACCACTATGAGATGGGGATGCGG	h10_Bs_R
TTGTAAGGGAAGAAAAAGTACCGTTCGAATAGGGCGGTACCTTGACAGTACCTTACCAGA	h17_Bs_F
GTACCGTTCGAATAGGGCGGTACCTTGACAGTACCTTACCAGA	h17_Bs_R
ACCTTAGAGTGCCCAACTGAATGCTGGCAACTAAGATCAAGGGTTGCGTTCGTTGCG	h39_Bs_F
ATCTTAGTTGCCAGCATTCAGTTGGGCACTCTAAGGTGACTGCTAGTGTAAGCTAGA	h39_Bs_R
GCCGCTCTTTACCGAAGTAAATCGCTCAACTTGCATGTATTAGGCATGCC	h6_Cd_F
GTTGAGCGATTTACTTCGGTAAAGAGCGGCGAACGGGTGAGTAACACGTA	h6_Cd_R
ATCTTATTATCGCATGAGAAAAGATCAAAAGGTGAGCCACTATGAGATGGGGATGCGGCG	h10_Cd_F
GGTGAGCCACTATGAGATGGGGATGCGG	h10_Cd_R
TCTGTTGTAAGGGAAGAAAAAGATAATGACCTTGACAGTACCTTACCAGAAAGCCACGGC	h17_Cd_F
GATAATGACCTTGACAGTACCTTACCAGA	h17_Cd_R

TCTCTAGAGTGCCCAACTTAATGATGGCAACTAAAGACAAGGGTTGCGTTCGTTGCG	h39_Cd_F
TCTTTAGTTGCCATCATTAAGTTGGGCACTCTAGAGAGACTGCTAGTGTAAGCTAGA	h39_Cd_R
GCCACTCGTCAGCAAAGAAGCAAGCTTCTTCCTGTTACCGTTCGACTTGCATGTATTAGGCATGCC	h6_Ec_F
GTCGAACGGTAACAGGAAGAAGCTTGCTTCTTTGCTGACGAGTGGCGAACGGGTGAGTAACACGTA	h6_Ec_R
CATGAGAAAAGATCAAAAGAGGGGGACCTTCGGGCCTCTTACTATGAGATGGGGATGCGG	h10_Ec_F
GAGGGGGACCTTCGGGCCTCTTACTATGAGATGGGGATGCGG	h10_Ec_R
TTGTAAGGGAAGAAAAAGAGTAAAGTTAATACCTTTGCTCCTTGACAGTACCTTACCAGA	h17_Ec_F
GAGTAAAGTTAATACCTTTGCTCCTTGACAGTACCTTACCAGA	h17_Ec_R
TCCTTTGAGTTCCCGGCCGGACCGCTGGCAACAAAGGACAAGGGTTGCGTTCGTTGCG	h39_Ec_F
TCCTTTGTTGCCAGCGGTCCGGCCGGGAACTCAAAGGAGACTGCTAGTGTAAGCTAGA	h39_Ec_R
GGTAAGACTAATTAAAGATGGTAGTGAATATAAAATGTCTAAAAG	Cas9_donor_F
GAACAATCTATATTCAAAGATGTGTGACGATCTCTG	Cas9_donor_R

Supplementary Table 3. Sequences of ultramers used for *rrs* engineering.

T1 and T6 ultramers were used to generate gRNAs for replacing the native *rrs* with the *ura3* cassette while gRNAs resulting from T9 and T10 ultramers were used for replacing the ura3 cassette with wild-type *rrs* or engineered *rrs**. Target sequences are indicated in red.

F_T1	TAATACGACTCACTATAGGTATCCGTACGGGAACGTGGTTTTAGAGCTAGAAATAGCAAGTTAAAATAAGGCTAGTCCGTTATCAACTTGAAAAAGTGGCACCGAGTCGGTGCTTTTTTT
R_T1	AAAAAAAGCACCGACTCGGTGCCACTTTTTCAAGTTGATAACGGACTAGCCTTATTTTAACTTGCTATTTCTAGCTCTAAAACCACGTTCCCGTACGGATACCTATAGTGAGTCGTATTA
F_T6	TAATACGACTCACTATAGCATACTGAGCATAATAAACGTTTTAGAGCTAGAAATAGCAAGTTAAAATAAGGCTAGTCCGTTATCAACTTGAAAAAGTGGCACCGAGTCGGTGCTTTTTTT
R_T6	AAAAAAAGCACCGACTCGGTGCCACTTTTTCAAGTTGATAACGGACTAGCCTTATTTTAACTTGCTATTTCTAGCTCTAAAACGTTTATTATGCTCAGTATGCTATAGTGAGTCGTATTA
F_T9	TAATACGACTCACTATACATAGTTAAGCCAGCCCAACGTTTTAGAGCTAGAAATAGCAAGTTAAAATAAGGCTAGTCCGTTATCAACTTGAAAAAGTGGCACCGAGTCGGTGCTTTTTTT
R_T9	AAAAAAAGCACCGACTCGGTGCCACTTTTTCAAGTTGATAACGGACTAGCCTTATTTTAACTTGCTATTTCTAGCTCTAAAACGTTGGGCTGGCTTAACTATGTATAGTGAGTCGTATTA
F_T10	TAATACGACTCACTATACACAGATGCGTAAGGAGGTGGTTTTAGAGCTAGAAATAGCAAGTTAAAATAAGGCTAGTCCGTTATCAACTTGAAAAAGTGGCACCGAGTCGGTGCTTTTTTT
R_T10	AAAAAAAGCACCGACTCGGTGCCACTTTTTCAAGTTGATAACGGACTAGCCTTATTTTAACTTGCTATTTCTAGCTCTAAAACCACCTCCTTACGCATCTGTGTATAGTGAGTCGTATTA

Supplementary Table 4. Diagnostic primers used to confirm the integrity of the *M. mycoides* genome through multiplex PCR.

When the genome is intact, seven bands, 204bp, 256bp, 301bp, 408bp, 515bp, 612bp and 724bp, are obtained after the diagnostic multiplex PCR.

Sequence	Name
GTAGAACATGTTGCAGATAAG	MPCR9-2F
TGATGGTGCATAACGTAATC	MPCR9-2R
ATTTAGTTGCTGATTGAGTTTG	MPCR9-3F
CTAGGACCAGTGGCTGC	MPCR9-3R
AGTTGACTGAAATTCTCAGTC	MPCR9-4F
AAGATCGTTTAGCTAAAGGAC	MPCR9-4R
TGGTTATATAATACTTGGAGTG	MPCR9-5F
GCAGGTTTTCCTCCTCTAC	MPCR9-5R
CATGATTGATATGTTTAAAGATGC	MPCR9-6F
TGCAAAGACTAAAGTATATGTG	MPCR9-6R
TTGATGTATCTCACTTATTTAGA	MPCR9-7F
CAACAACTAGAGATTTAAACAG	MPCR9-7R
GCTTGTTCAACATTATAAGCTC	MPCR9-8F
TTGATGGTGACTCTAATAATACT	MPCR9-8R

Supplementary Table 5. Primers used to confirm genome-editing event through multiplex PCR.

In the presence of an editing event, 353bp and 474bp bands would be observed, while a ~1.8kb amplicon would be obtained if the *ura3* cassette is not replaced by *rrs**.

Sequence	Name
GCAGGTAGTCACGTCCTTCTTCGG	FJ1-Diagn16S
GTCGCGGTGAATACGTTCTCGG	RJ1-Diagn16S
GCTTGGTTTAAAATTGAATGACATCTAGCAGTAGC	FJ2-Diagn16S
AAATTAATTAATGTTTGAGGTGGAGATCATCATGG	RJ2-Diagn16S

Sequences of the heterologous 16S rRNA genes used to substitute the entire wild-type *M. mycoides* 16S rRNA gene

E. coli

AGATAAGGAGGTGATCCAACCGCAGGTTCCCCTACGGTTACCTTGTTACGACTTCACCCCAGTCATGAATCACAAAG
TGGTAAGCGCCCTCCCGAAGGTTAAGCTACCTACTTCTTTTGCAACCCACTCCCATGGTGTGACGGGCGGTGTGTAC
AAGGCCCGGGAACGTATTCACCGTGGCATTCTGATCCACGATTACTAGCGATTCCGACTTCATGGAGTCGAGTTGCA
GACTCCAATCCGGACTACGACGCACTTTATGAGGTCCGCTTGCTCTCGCGAGGTCGCTTCTCTTTGTATGCGCCATT
GTAGCACGTGTGTAGCCCTGGTCGTAAGGGCCATGATGACTTGACGTCATCCCCACCTTCCTCCAGTTTATCACTGG
CAGTCTCCTTTGAGTTCCCGGCCGGACCGCTGGCAACAAAGGATAAGGGTTGCGCTCGTTGCGGGACTTAACCCAAC
ATTTCACAACACGAGCTGACGACAGCCATGCAGCACCTGTCTCACGGTTCCCGAAGGCACATTCTCATCTCTGAAAA
CTTCCGTGGATGTCAAGACCAGGTAAGGTTCTTCGCGTTGCATCGAATTAAACCACATGCTCCACCGCTTGTGCGGG
CCCCCGTCAATTCATTTGAGTTTTAACCTTGCGGCCGTACTCCCCAGGCGGTCGACTTAACGCGTTAGCTCCGGAAG
CCACGCCTCAAGGGCACAACCTCCAAGTCGACATCGTTTACGGCGTGGACTACCAGGGTATCTAATCCTGTTTGCTC
CCCACGCTTTCGCACCTGAGCGTCAGTCTTCGTCCAGGGGGCCGCCTTCGCCACCGGTATTCCTCCAGATCTCTACG
CATTTCACCGCTACACCTGGAATTCTACCCCCCTCTACGAGACTCAAGCTTGCCAGTATCAGATGCAGTTCCCAGGT
TGAGCCCGGGGATTTCACATCTGACTTAACAAACCGCCTGCGTGCGCTTTACGCCCAGTAATTCCGATTAACGCTTG
CACCCTCCGTATTACCGCGGCTGCTGGCACGGAGTTAGCCGGTGCTTCTTCTGCGGGTAACGTCAATGAGCAAAGGT
ATTAACTTTACTCCCTTCCTCCCCGCTGAAAGTACTTTACAACCCGAAGGCCTTCTTCATACACGCGGCATGGCTGC
ATCAGGCTTGCGCCCATTGTGCAATATTCCCCACTGCTGCCTCCCGTAGGAGTCTGGACCGTGTCTCAGTTCCAGTG
TGGCTGGTCATCCTCTCAGACCAGCTAGGGATCGTCGCCTAGGTGAGCCGTTACCCCACCTACTAGCTAATCCCATC
TGGGCACATCCGATGGCAAGAGGCCCGAAGGTACCCCTCTTTGGTCTTGCGACGTTATGCGGTATTAGCTACCGTTT
CCAGTAGTTATCCCCCTCCATCAGGCAGTTTCCCAGACATTACTCACCCGTCCGCCACTCGTCAGCAAAGAAGCAAG
CTTCTTCCTGTTACCGTTCGACTTGCATGTGTTAGGCCTGCCGCCAGCGTTCAATCTGAGCCATGATCAAACTCTTC
AATTT

Streptomyces scabiei

AGAAAGGAGGTGATCCAGCCGCACCTTCCGGTACGGCTACCTTGTTACGACTTCGTCCCAATCGCCAGTCCCACCTT
CGACAGCTCCCTCCCTTACGGGTTGGGCCACCGGCTTCGGGTGTTACCGACTTTCGTGACGTGACGGGCGGTGTGTA
CAAGGCCCGGGAACGTATTCACCGCAGCAATGCTGATCTGCGATTACTAGCAACTCCGACTTCATGGGGTCGAGTTG
CAGACCCCAATCCGAACTGAGACAGGCTTTTTGAGATTCGCTCCGCCTCACGGCTTCGCAGCTCATTGTACCTGCCA
TTGTAGCACGTGTGCAGCCCAAGACATAAGGGGCATGATGACTTGACGTCGTCCCCACCTTCCTCCGAGTTGACCCC
GGCAGTCTCCTGTGAGTCCCCATCACCCCGAAGGGCATGCTGGCAACACAGAACAAGGGTTGCGCTCGTTGCGGGAC
TTAACCCAACATCTCACGACACGAGCTGACGACAGCCATGCACCACCTGTACACCGACCACAAGGGGGCGACCATCT
CTGGCCGTTTCCGGTGTATGTCAAGCCTTGGTAAGGTTCTTCGCGTTGCGTCGAATTAAGCCACATGCTCCGCTGCC
TGTGCGGGCCCCGTCAATTCCTTTGAGTTTTAGCCTGCGGCCGTACTCCCCAGGCGGGGAACTTAATGCGTTAGCTG
CGGCACCGACGACGTGGAATGTCGCCAACACCTAGTTCCCACCGTTTACGGCGTGGACTACCAGGGTATCTAATCCT
GTTCGCTCCCCACGCTTTCGCTCCTCAGCGTCAGTAATGGCCCAGAGATCCGCCTTCGCCACCGGTGTTCCTCCTGA
TATCTGCGCATTTCACCGCTACACCAGGAATTCCGATCTCCCCTACCACACTCTAGTCTGCCCGTATCGAATGCAGA
CCCGGGGTTAAGCCCCGGGCTTTCACATCCGACGCGACAGACCGCCTACGAGCTCTTTACGCCCAATAATTCCGGAC
AACGCTCGCGCCCTACGTATTACCGCGGCTGCTGGCACGTAGTTAGCCGGCGCTTCTTCTGCAGGTACCGTCACTTT
CGCTTCTTCCCTGCTGAAAGAGGTTTACAACCCGAAGGCCGTCATCCCTCACGCGGCGTCGCTGCATCAGGCTTTCG
CCCATTGTGCAATATTCCCCACTGCTGCCTCCCGTAGGAGTCTGGGCCGTGTCTCAGTCCCAGTGTGGCCGGTCGCC
CTCTCAGGCCGGCTACCCGTCGTCGCCTTGGTGAGCCGTTACCTCACCAACAAGCTGATAGGCCGCGGGCTCATCCT
TCACCGCCGGAGCTTTCCACACTCATCGGATGCCCGAGAGTGTCGTATCCGGTATTAGACCCCGTTTCCAGGGCTTG
TCCCAGAGTGAAGGGCAGATTGCCCACGTGTTACTCACCCGTTCGCCACTAATCCCCACCGAAGTGGTTCATCGTTC
GACTTGCATGTGTTAAGCACGCCGCCAGCGTTCGTCCTGAGCCAGGATCAAACTCTCCGTGAATGTTT

Bacillus subtilis

AGAAAGGAGGTGATCCAGCCGCACCTTCCGATACGGCTACCTTGTTACGACTTCACCCCAATCATCTGTCCCACCTT
CGGCGGCTGGCTCCTAAAAGGTTACCTCACCGACTTCGGGTGTTACAAACTCTCGTGGTGTGACGGGCGGTGTGTAC
AAGGCCCGGGAACGTATTCACCGCGGCATGCTGATCCGCGATTACTAGCGATTCCAGCTTCACGCAGTCGAGTTGCA
GACTGCGATCCGAACTGAGAACAGATTTGTGGGATTGGCTTAACCTCGCGGTTTCGCTGCCCTTTGTTCTGTCCATT
GTAGCACGTGTGTAGCCCAGGTCATAAGGGGCATGATGATTTGACGTCATCCCCACCTTCCTCCGGTTTGTCACCGG
CAGTCACCTTAGAGTGCCCAACTGAATGCTGGCAACTAAGATCAAGGGTTGCGCTCGTTGCGGGACTTAACCCAACA
TCTCACGACACGAGCTGACGACAACCATGCACCACCTGTCACTCTGCCCCCGAAGGGGACGTCCTATCTCTAGGATT
GTCAGAGGATGTCAAGACCTGGTAAGGTTCTTCGCGTTGCTTCGAATTAAACCACATGCTCCACCGCTTGTGCGGGC
CCCCGTCAATTCCTTTGAGTTTCAGTCTTGCGACCGTACTCCCCAGGCGGAGTGCTTAATGCGTTAGCTGCAGCACT
AAGGGGCGGAAACCCCCTAACACTTAGCACTCATCGTTTACGGCGTGGACTACCAGGGTATCTAATCCTGTTCGCTC
CCCACGCTTTCGCTCCTCAGCGTCAGTTACAGACCAGAGAGTCGCCTTCGCCACTGGTGTTCCTCCACATCTCTACG
CATTTCACCGCTACACGTGGAATTCCACTCTCCTCTTCTGCACTCAAGTTCCCCAGTTTCCAATGACCCTCCCCGGT
TGAGCCGGGGGCTTTCACATCAGACTTAAGAAACCGCCTGCGAGCCCTTTACGCCCAATAATTCCGGACAACGCTTG
CCACCTACGTATTACCGCGGCTGCTGGCACGTAGTTAGCCGTGGCTTTCTGGTTAGGTACCGTCAAGGTACCGCCCT
ATTCGAACGGTACTTGTTCTTCCCTAACAACAGAGCTTTACGATCCGAAAACCTTCATCACTCACGCGGCGTTGCTC
CGTCAGACTTTCGTCCATTGCGGAAGATTCCCTACTGCTGCCTCCCGTAGGAGTCTGGGCCGTGTCTCAGTCCCAGT
GTGGCCGATCACCCTCTCAGGTCGGCTACGCATCGTCGCCTTGGTGAGCCGTTACCTCACCAACTAGCTAATGCGCC
GCGGGTCCATCTGTAAGTGGTAGCCGAAGCCACCTTTTATGTTTGAACCATGCGGTTCAAACAACCATCCGGTATTA
GCCCCGGTTTCCCGGAGTTATCCCAGTCTTACAGGCAGGTTACCCACGTGTTACTCACCCGTCCGCCGCTAACATCA
GGGAGCAAGCTCCCATCTGTCCGCTCGACTTGCATGTATTAGGCACGCCGCCAGCGTTCGTCCTGAGCCAGGATCAA
ACTCTCCGATAAA

Clostridium acetobutylicum

AGAAAGGAGGTGATCCAGCCGCAGGTTCTCCTACGGCTACCTTGTTACGACTTCACCCCAATTATCAACCCCACCTT
CGACCGCTGGTTCCAAAAGGTTACCTCACGGGCTTCGGGTGTTGCCGACTCTCATGGTGTGACGGGCGGTGTGTACA
AGACCCGGGAACGTATTCACCGCGACATTCTGATTCGCGATTACTAGCAACTCCGGCTTCATGTAGGCGAGTTTCAG
CCTACAATCCGAACTGGGATGGGGTTTTGAGTTTTGCTCCACCTTGCGGTATTGCATCTTTTTGTCCCCACCATTGT
AGCACGTGTGTAGCCCTAGACATAAGGGGCATGATGATTTGACGTCATCCCCACCTTCCTCCCGGTTAACCCGGGCA
GTCTCACTAGAGTGCTCAACTAAATGTTAGCAACTAATGATAAGGGTTGCGCTCGTTGCGGGACTTAACCCAACATC
TCACGACACGAGCTGACGACAACCATGCACCACCTGTCATCCTGTCCCCGAAGGGACTTCATCCATTACGGACTAAT
TCAGGAGATGTCAAGTCTAGGTAAGGTTCTTCGCGTTGCTTCGAATTAAACCACATGCTCCGCTGCTTGTGCGGGTC
CCCGTCAATTCCTTTGAGTTTTAATCTTGCGACCGTACTTCCCAGGCGGAATACTTATTGTGTTAACTGCGGCACAG
AAGGAGTCGATACCTCCTACACCTAGTATTCATCGTTTACGGCGTGGACTACCAGGGTATCTAATCCTGTTTGCTCC
CCACGCTTTCATGCCTCAGCGTCAGTTACAGTCCAGAAGGCCGCCTTCGCCACTGGTATTCTTCCTAATCTCTACGC
ATTTCACCGCTACACTAGGAATTCTGCCTTCCTCTCCTGCACTCCAGACATCCAGTTTGAAATGCAGCCCCCAAGTT
AAGCCCGGGGATTTCACATCTCACTTAAATATCCGCCTACACATCCTTTACGCCCAGTAAATCCGGACAACGCTTGC
CACCTACGTATTACCGCGGCTGCTGGCACGTAGTTAGCCGTGGCTTCCTCCTATGGTACCGTCATTATCGTCCCATA
AGACAGAGTTTTACGATCCGAAGACCTTCATCACTCACGCGGCGTTGCTGCATCAGGGTTTCCCCCATTGTGCAATA
TTCCCCACTGCTGCCTCCCGTAGGAGTCTGGACCGTGTCTCAGTTCCAATGTGGCCGATCACCCTCTCAGGTCGGCT
ACGCATCGAAGCCTTGGTGAGCCGTTACCTCACCAACAAGCTAATGCGCCGCGGGTCCATCTCATAGCGAATAAATC
CTTTGGCTCAAGAATCATGCGATTCTCGAGTATTATGCGGTATTAATCTTCCTTTCGAAAGGCTATTCCCCACTATG
AGGTAGGTTACCCACGTGTTACTCACCCGTCCGCCGCTGGGAACCGAAGTTCCCCGCTCGACTTGCATGTGTTAAGC
ACGCCGCCAGCGTTCGTCCTGAGCCAGGATCAAACTCTCAATTT

Acholeplasma laidlawii

AGAAAGGAGGTGATCCATCCCCACGTTCCCGTAGGGATACCTTGTTACGACTTAACCCCAATCATGGACCCTACCTT
AGACGGCTCCCTCCCTTTCGGGTTAGGCCACCGGCGTTGGGTATTGCCCACTTTCGTGGTTTGACGGGCGGTGTGTA
CAAACCCCGAGAACGTATTCACCGCAACATGCTGATTTGCGATTACTAGCGATTCCGACTTCATGGAGTCGAGTTGC
AGACTCCAATCCGAACTGAGACTGTTTTTGTGAGTTTCGCTCCAGGTCACCCTATCGCTTCTCTTTGTTCCAGCCAT
TGTATCACGTTTGTAGCCCAGGTCATAAGGGGCATGATGATTTGACGTCATCCCCACCTTCCTCCAATTTATCACTG
GCAGTCTCGCTAGAGTCCCCAACTTAATGATGGTAACTAGCAATAAGGGTTGCGCTCGTTGCGGGACTTAACCCAAC
ATCTCACGACACGAGCTGACGACAACCGTGCACCACCTGTACATCTGTTAGCCTCCGAACTTATTTCTAAGCCTTTG
CAGAGTATGTCAAGACCTGGTAAGGTTTTTCGTGTATCTTCGAATTAAACAACATGATCCACCGCTTGTGCGGGGTC
CCGTCAATTCCTTTGAGTTTCATACTTGCGTACGTACTACTCAGGCGGAGAACTTAATGCGTTAACTGCAGCACTGA
CCTTTTGGCCAACACTTAGTTCTCATCGTTTACGGCGTGGACTACCAGGGTATCTAATCCTGTTTGCTCCCCACGCT
TTCGTGCATCAGTGTCAGTATAGACCCAGCAAGCCGCCTTCGCCACTGGTGTTCCTCCATATATTTACGCATTTTAC
CGCTACACATGGAATTCCACTTGCCTCTGTCTCACTCTAGTTATATAGTTTTCATAGCCTCACAGCGTTAAGCACTG
CACTTACACCACAAACTTTTATAACCACCTACGCACCCTTTACGCCCAGTAAATCCGGATAACGCTCGCCCCCTATG
TATTACCGCGGCTGCTGGCACATAGTTAGCCGGGGCTTATTCATATGGTACCGTCAATTTTTAATTTTTCTTCCCAT
ATAAAAGAACTTTACATACCGAAGTACTTCGTCGTTCACGCGGCGTTGCTCGGTCAGGGTTTCCCCCATTGCCGAAA
ATTCCCTACTGCTGCCTCCCGTAGGAGTTTGGGCCGTTCTCAGTCCCAATGTGGCCGGTAGACCTCTCAGTCCGGCT
ACGCATCATCGTCTTGGTAGGCTTTTACCCCACCAACTAACTAATGCGCCGCAGACCCCTCTTAAACCGATAAATCT
TTAAATGTGTTTTTTTCATGCACACATCCTATCCAGTATTAGCTCCAGTTTCCCGGAGTTATCCTCGAGTTAAAGGT
AGGTTATCTACGTGTTACTCACCCGTTCGCCACTAAGCATCCGAAGATGCTTCGTTCGACTTGCATGTATTAGGCAC
GCCGCCAGCGTTCATCCTGAGCCAGGATCAAACTCTCCATATA

Spiroplasma taiwanense

AAAATGAGAGTTTGATCCTGGCTCAGGATGAACGCTGGCGGCATGCCTAATACATGCAAGTCGAACGGGGTGCTTGC
ACCCAGTGGCGAACGGGTGAGTAACACGTATCTAATCTACCTTTTAGTGGGGGATAACAGTTGGAAACGACTGCTAA
TACCGCATACGACACCATTATGGCATCAGAAGGTGTTAAAAGGCCCGTTTGGGTCGCTAGAAGATGAGGATGCGGCG
TATTAGTTAGTAGGTGAGGTAATTGCTCACCTAGGCGATGATACGTAGCCGAACTGAGAGGTTGATCGGCCACATTG
GGACTGAGATACGGCCCAGACTCCTACGGGAGGCAGCAGTAGGGAATTTTTCACAATGGACGAAAGTCTGATGAAGC
AATGCCGCGTGAGTGATGACGGTCTTCGGATTGTAAAGCTCTGTTGTAAGGGAAGAAATGCTAGAAAAGGAAATGTT
TTTAGTTTGACGGTACCTTACCAGAAAGCCACGGCTAACTATGTGCCAGCCGCCGCGGTAATACATAGGTGGCAAGC
GTTATCCGGATTTATTGGGCGTATAGGGTGCGTAGGCGGTTAGTTAAGTTTGAGGTTAAAGCCCGGAGCTCAACTCC
GGTTCGCCTTGAAAACTGGCTAACTAGAATGCAGGAGAGGTAGATGGAATTCCATGTGTAGCGGTGAAATGCGTAGA
TATATGGAGGAACACCAGTGGCGTAGGCGGTCTACTGGCCTGTGATTGACGCTGAGGCACGAAAGCGTGGGGAGCAA
ATAGGATTAGATACCCTAGTAGTCCACGCCGTAAACGTTGAGTACTAAGTGTCGGCATTTTGTCGGTGCTGCAGCTA
ACGCATTAAGTACTCCGCCTGAGTAGTATGCTCGCAAGAGTGAAACTCAAAGGAATTGACGGGGACCCGCACAAGTG
GTGGAGCATGTGGTTTAATTCGAAGCAACGCGAAGAACCTTACCAGGGCTTGACATCCAGTGCAAAGCTACAGAGAT
GTAGTGGAGGCTAACATTGAGACAGGTGGTGCATGGTTGTCGTCAGCTCGTGCCGTGAGGTGTTGGGTTAAGTCCCG
CAACGAGCGCAACCCCTATCATTAGTTACTAACATTCAGTTGAGGACTCTAATGAGACTGCTAGTGTAAGCTAGAGG
AAGGTGGGGATGACGTCAAATCATCATGCCCCTTATGTCCTGGGCTACACACGTGCTACAATGGCTGATACAAAGAG
TTGCAATCCTGCGAGGGGGAGCTAATCTCAAAAAGTCAGTCTCAGTTCGGATTGAAGTCTGCAACTCGACTTCATGA
AGCCGGAATCACTAGTAATCGCGAATCAGCAACGTCGCGGTGAATACGTTCTCGGGTCTTGTACACACCGCCCGTCA
CACCATGAGAGTTGGTAATACCAGAAGCACGTATCTTAACCGTAAGGAGGGAGCGTACCAAGGTAGGATTAGCGATT
AGGGTGAAGTCGTAACAAGGTATCCGTACGGGAACGTGCGGATGGATCACCTCCTTTCT

Mycoplasma capricolum

AGAAAGGAGGTGATCCATCCGCACGTTCCCGTACGGATACCTTGTTACGACTTCACCCCAATCGCTAGTCCTACCTT
GGGAAGCGCTCTCCAAATGGTTAAGCTACCTACTTCTGGTATTACCAACTCTCATGGTGTGACGGGCGGTGTGTACA

AGACCCGAGAACGTATTCACCGCGACATAGCTGATTCGCGATTACTAGTGATTCCGGCTTCATGAAGTCGAGTTGCA
GACTTCAATCCGAACTGAGACTGGTTTTTTGAGATTAGCTCCCCTTCACAGGATTGCAACTCTTTGTACCAGCCATT
GTAGCACGTGTGTAGCCCAGGACATAAGGGGCATGATGATTTGACGTCATCCCCACCTTCCTCTAGCTTACACTAGC
AGTCTCGTTAGAGTTCTCAACTTAATGTTAGTAACTAACGACAAGGGTTGCGTTCGTTGCGGGACTTAACCCAACAC
CTCACGGCACGAACTGACGACAACCATGCACCACCTGTCTCAATGTTAACCTCTACTATATCTCTATAGCTTTGCAC
TGGATGTCAAGCCCTGGTAAGGTTCTTCGTGTTGCTTCGAATTAAACCACATGCTCCACCACTTGTGCGGGTCCCCG
TCAATTCCTTTGAGTTTCACTCTTGCGAGCATACTACTCAGGCGGAGTACTTAATGCGTTAGCTGCAGCGCTGAGTT
ACCCCAACACTTAGTACTCATCGTTTACGGCGTGGACTACTAGGGTATCTAATCCTATTTGCTCCCCACGCTTTCGT
GCCTCAGCGTCAATAACAAGCCAGTAAGCCGCTTTCGCCACAGGTGTTCTTCCATATATCTACGCATTTCACCGCTA
CACATGGAATTCCGCTTACCTCTCTTGCATTCTAGTAAAACAGTCTTCAAGGCGAACCGGAGTTGAGCTCCGGACTT
TAACCTCAAACTTGCAAAACCGCCTACGCACCCTATACGCCCAATAAATCCGGATAACGCTTGCCACCTATGTATTA
CCGCGGCTGCTGGCACATAGTTAGCCGTGGCTTTCTGGTAAGGTACTGTCAAGATAAAGTCATTTCCTACTCTATTT
TTTCTTCCCTTACAACAGAGCTTTACAACCCGAAGGCCGTCATCACTCACGCGGCATTGCTTCATCAGACTTTCGTC
CATTGTGAAAAATTCCCTACTGCTGCCTCCCGTAGGAGTCTGGGCCGTATCTCAGTCCCAATGTGGCCGATCAACCT
CTCAGTTCGGCTACGTATCATCGCCTAGGTGGGCTATTATCTCACCTACTAGCTAATACGCCGCATCCCCATCTCAT
AGTGAACCAAACGGTTCTTTTGATCTTTTCTCATGCGATAATAAGATCTACATGCGGTATTATCTTTCGTTTCCAAA
AGTTATCCCCCGCTATAAGGTAGGTTAGATACGTGTTACTCACCCGTTCGCCACTGAGGTGCAAGCACCCCCGTTCG
ACTTGCATGTATTAGGCATGCCGCCAGCGTTTATCCTGAGCCAGGATCAAACTCTCATTTT

Mesoplasma florum

AGAAAGGAGGTGATCCATCCGCACGTTCCCGTACGGATACCTTGTTACGACTTCACCCCAATCGCTAGTCCTACCTT
GGGACGCGCCTTCCTTACGGTTAGGAAACGTACTTCTGGTATTACCAACTCTCGTGGTTTGACGGGCGGTGTGTACA
AGACCCGAGAACGTATTCACCGCGACATAGCTGATTCGCGATTACTAGTGATTCCGGCTTCATGAAGTCGAGTTGCA
GACTTCAATCCGAACTGAGACCGACTTTTTGAGATTAGCTCCCCCTCGCGAGATTGCGACTCTTTGTATCGGCCATT
GTAGCACGTGTGTAGCCCAGGACATAAGGGGCATGATGATTTGACGTCATCCCCACCTTCCTCTAGCTTACACTAGC
AGTCTCGTTAGAGTCCTCAACTTAATGTTAGTAACTAACGACAAGGGTTGCGTTCGTTGCGGGACTTAACCCAACAC
CTCACGGCACGAACTGACGACAACCATGCACCACCTGTATCAATGTTAACCTCCACTACATCTCTGTAGCTTTGCAC
TGTATGTCAAGCCCTGGTAAGGTTCTTCGTGTTGCTTCGAATTAAACCACATGCTCCACCACTTGTGCGGGTCCCCG
TCAATTCCTTTGAGTTTCACTCTTGCGAGCATACTACTCAGGCGGAGTACTTAATGCGTTAGCTGCAGCACTGAGGT
AATCCCCAATACTTAGTACTCAACGTTTACGGCGTGGACTACTAGGGTATCTAATCCTATTTGCTCCCCACGCTTTC
GTGCCTCAGCGTCAATAATCAGCCAGTAAGCCGCTTTCGCCACTGGTGTTCTTCCATATATCTACGCATTTTACCGC
TACACATGGAATTCCGCTTACCTCTCTGATATTCTAGTCCCGCAGTTTTCAAGGCGAACCGGAGTTGAGCTCCGGGC
TTTAACCTCAAACTTGCGAAACCGCCTACGCACCCTATACGCCCAATAAATCCGGATAACGCTTGCCACCTATGTAT
TACCGCGGCTGCTGGCACATAGTTAGCCGTGGCTTTCTGGTAAGGTACCGTCAAGATAAGAGCATTTCCTCTCCTAT
GTTTTCTTCCCTTACAACAGAGCTTTACAACCCGAAGGCCGTCATCACTCACGCGGCATTGCTTCATCAGACTTTCG
TCCATTGTGAAAAATTCCCTACTGCTGCCTCCCGTAGGAGTCTGGGCCGTATCTCAGTCCCAATGTGGCCGATCAAC
CTCTCAGTTCGGCTACGTATCATCGCCTAGGTGGGCCTTTACCCCGCCTACTAGCTAATACGCCGCATCCTCATCTT
CTAGTGATCCAAACGGATCTTTGAATGTTTTCTCATGCGATAATAACATCCACATGCGGTATTACCTTTCGTTTCCA
AAAGTTATCCCCCGCTAGAAGGTAGATTAGATACGTGTTACTCACCCGTTCGCCACTGAGGTGCAAGCACCTCCGTT
CGACTTGCATGTATTAGGCATGCCGCCAGCGTTTATCCTGAGCCAGGATCAAACTCTCATTTT

Chloroflexus aggregans

AGAAAGGAGGTGATCCAGCCGCACCTTCCGGTACGGCTACCTTGTTACGACTTCGTCCCAGTCGCGGCTCCTGCCCT
CGGCCGCTGCCTCCCCTGACGGGGTTAGCGCACGGACTTCAAGCATTAGCCACTCCCATGACGTGACGGGCGGTGTG
TACAAGGCCCGGGAACGTATTCACCGCGCCATGGCTGATACGCGGTTACTAGCAACTCCGCCTTCACGGGGGCGAGT
TGCAGCCCCCGATCTGCACTGAGACCACGTTTGGCGATTAGCGCCGCCTTGCGGCGTGGCAACGCATTGTCGTGGCC
ATTGTAGCGTGTGTGTCGCCCCGGACGTCAGGGCCATGCTGACTTGACGTCATCCGCGCCTTCCTCCGCCGGTCTCG
GCGGCAGTCTCGGTAGACACCGGTAACTACCGACGCGGGTTGCGCTCGTTGCGGGACTGAACCCAACATCTCACGAC

ACGAGCTGACGACAGCCATGCAGCACCTGTGACGCACCCTCAAAGGCCGCGCTGTTTCCAGCACCTGCAGCGTCATG
TCAAGCCCGGGTAAGGTTCTTCGGGTTGCGTCGAATTAAACCACACGCTCCGCTGCTTGTGCGGGCCCCCGTCAATT
CCTTTGAGTTTTAAGCTTGCGCTCGTAGTCCCCAGGCGGCCGGCTTACCGCGTTAGCTAAGGCACCCGGCACGCTCA
CGCGCGCCAAACGCCGAGCCGGCATCGTTTACGGCGTGGACTACCCGGGTATCTAATCCGGTTTGCTCCCCACGCGG
TCGCGCCGCAGCGTCAGGTCGAGCCCAGGTGGCCGACTGCTCCACCGGCGTTCCTCCGGATCTCTACGCATTTCACC
GCTCCACCCGGAATTCCACCACCCTCTGCTCGCCTCCAGCCTGCTCGTATCGGCGCGCCTCGCCCCGTTAAGCGGGG
CGCTTTCAGCGCCGACGCAGCAGACCGCCTCCGCGCCCTTTACGCCCAGTAACTCCGGACAACGCTTGCCCCCTCTG
TCTTACCGCGGCTGCTGGCACAGAGTTAGCCGGGGCTGCTTCTGGTGGTACCGTCAGTCTCGTCCCACCCGAAAGGA
GTTTACAACCCGAAGGCCGTCGTCCTCCACGCGGCGTTGCTCCCTCAGGCGTGCGCCCATTGGGGAAAATTCCTTGC
TGCTGCCTCCCGTAGGAGTCTGGGCCGTGTCGCAGTCCCAGTCTGCCCGGCCGTCCTCTCAGACCGGGTACCCGTCA
GTGGCTTGGTGCGCCGTTACCACACCAACTACCTGATGGGCCGCAGGCCCGTCCCGGAGCGCCGTATGGCTTTCCTC
CGTAGAGCGTATGCGGGATTGTCCCGTCTTTCGACGGGGTATCCCCCACTCCGGGGGTGGTGACCCACGTGTTCCTC
AGCCGTTCGCCACGCAGCCGGCACTTGGCCGCTGCGTTCGACTTGCATGCATTAGGCACGCCGCCAGCGTTCGTCCT
GAGCCAGGATCAAACTCTCATTTT

Deinococcus radiodurans

AGAAAGGAGGTGATCCAACCGCACCTTCCGGTACAGTTACCTTGTTACGACTTCACCCCAGTCATAAACCACAGTCT
AGACGCCTGCCGTTAAGCTCCCGGCGGTTTCAACTGCAATCTACTCCCATGGTGTGACGGGCGGTGTGTACAAGGCC
CGGGAACGTATTCACCGCGGTATGCTGACCCGCGATTACTAGCGATTCCAACTTCACGGAGTCGAGTTGCAGACTCC
GATCTGAACTGGGGATAGGTTTTAGCGATTCGCTTACCCTCGCGGGTTTGCTGCGCGTTGTCCTACCCATTGTAGCA
CGTGTGTCGCCCAGGACGTAAGGACCATGCTGACTAGACGTCATCCCCGCCTTCCTCCTACTTTCATAGGCAGTCCC
TCTAGAGTGTCCAACTGAATGCTGACAACTAAAGGCAAGGGTTGCGCTCGTTGCGGGACTTAACCCAACATCTCACG
ACACGAGCTGACGACAGCCATGCAGCACCTGTGTCTAGGTTCCCCGAAGGGCACCTCTGCATCTCTGCAAAGTTCCT
AGCATGTCAAGACCTGGTAAGGTTCTTCGCGTTGCTTCGAATTAAACCACATGCTCCACCGCTTGTGCGGGCCCCCG
TCAATTCCTTTGAGTTTCAACCTTGCGGCCGTACTTCCCAGGCGGTACGTTTATCGCGTTAGCTTCGCCAAGCACAG
CATCCTGCGCTTAGCCAACGTACATCGTTTAGGGTGTGGACTACCCGGGTATCTAATCCGGTTTGCTCCCCACACTT
TCGCGCCTCAGCGTCACCTTCTGTCCAGTAACTTGCCTTCGCCATTGGTGTTCCTCCTGGTATCTACGCATTCCACC
GCTACACCAGGAATTCCAGTTACCTCTCCAGAGGTCAAGAAATCCAGTATCCAGTCCATCCCCGAGGTTGAGCCCCG
GTCTTTAAAACCAGACTTAAATTTCCGCCTACACGCCCTTTACGCCCAGTGATTCCGGGTAACGCTTGCACCCTCCG
TATTACCGCGGCTGCTGGCACGGAGTTAGCCGGTGCTATTACTCTGGTACCGTCATCTGCCCTAAGGCTCTTTCGTC
CCAGATTCAGAGGTTTACGATCCGAAAACCTTCATCCCTCACGCGGCGTCGCTCCATCAGGCTTGCGCCCATTGTGG
AAGATTCCTAACTGCTGCCTCCCGTAGGAGTGGGACCCGTGTCTCAGTGCCCCTGTGGCCGGCCACCCTCTCAGGCC
GGCTATCCGTCGTCGCCTTGGTAGGCCTTTACCCCACCAACCAGCTGATGGAACGCAACCCCATCCAGAAGCGATAA
ATCTTTAGTGATGCACCACGGTGCATCACCACATCACGTATTAGCGGACCTTTCGGCCAGTTATTCGTGACTTCTGG
GTAGGTCAGTTACGTGTTACTCACCCGTGCGCCACTCGGTCCGAAGACCGCGTTCGACTTGCATGTCTTAAGCACGC
CGCCAGCGTTCACCCTGAGCCAGGATCAAACTCTCCATAAA
